# Identification of adipocyte plasma membrane-associated protein as a novel modulator of human cytomegalovirus infection

**DOI:** 10.1371/journal.ppat.1007914

**Published:** 2019-07-29

**Authors:** Xiaohua Ye, Xun Gui, Daniel C. Freed, Zhiqiang Ku, Leike Li, Yuanzhi Chen, Wei Xiong, Xuejun Fan, Hang Su, Xi He, Richard R. Rustandi, John W. Loughney, Ningning Ma, Amy S. Espeseth, Jian Liu, Hua Zhu, Dai Wang, Ningyan Zhang, Tong-Ming Fu, Zhiqiang An

**Affiliations:** 1 Texas Therapeutics Institute, Brown Foundation Institute of Molecular Medicine, University of Texas Health Science Center at Houston, Texas, United States of America; 2 MRL, Merck & Co., Inc., Kenilworth, NJ, United States of America; 3 Wuya College of Innovation, Shenyang Pharmaceutical University, Shenyang, China; 4 Rutgers Medical School of New Jersey, Newark, NJ, United States of America; Tufts University, UNITED STATES

## Abstract

Human cytomegalovirus (HCMV) is a ubiquitous pathogen that can cause disability in newborns and serious clinical diseases in immunocompromised patients. HCMV has a large genome with enormous coding potential; its viral particles are equipped with complicated glycoprotein complexes and can infect a wide range of human cells. Although multiple host cellular receptors interacting with viral glycoproteins have been reported, the mechanism of HCMV infection remains a mystery. Here we report identification of adipocyte plasma membrane-associated protein (APMAP) as a novel modulator active in the early stage of HCMV infection. APMAP is necessary for HCMV infection in both epithelial cells and fibroblasts; knockdown of APMAP expression significantly reduced HCMV infection of these cells. Interestingly, ectopic expression of human APMAP in cells refractory to HCMV infection, such as canine MDCK and murine NIH/3T3 cells, promoted HCMV infection. Furthermore, reduction in viral immediate early (IE) gene transcription at 6 h post infection and delayed nucleus translocation of tegument delivered pp65 at 4 h post infection were detected in APMAP-deficient cells but not in the wildtype cells. These results suggest that APMAP plays a role in the early stage of HCMV infection. Results from biochemical studies of APMAP and HCMV proteins suggest that APMAP could participate in HCMV infection through interaction with gH/gL containing glycoprotein complexes at low pH and mediate nucleus translocation of tegument pp65. Taken together, our results suggest that APMAP functions as a modulator promoting HCMV infection in multiple cell types and is an important player in the complex HCMV infection mechanism.

## Author summary

Human cytomegalovirus (HCMV) is a prototypic β-herpesvirus that merits attention because of its ubiquitous prevalence and clinical significance among immature and immune compromised populations. Since its first isolation in 1956, there have been continuous efforts on understanding the infection mechanism of HCMV, which could serve as the basis for antiviral drugs and vaccines. Unfortunately, understanding of HCMV infection mechanisms and key host cellular factors involved in viral entry remain elusive, partly due to the complexity of HCMV. We report here identification of a type II membrane protein, adipocyte plasma membrane associated protein (APMAP), as a novel modulator promoting HCMV infection. We demonstrated that APMAP is both necessary and sufficient for HCMV infection of multiple cell types. APMAP may function through the interaction with viral gH/gL-containing glycoprotein complexes at low pH and mediate nuclear translocation of pp65, a viral tegument protein. Taken together, our results indicate that APMAP serves as an augmenting modulator of HCMV at the early stage of viral infection.

## Introduction

Human cytomegalovirus (HCMV), also known as human herpesvirus 5 (HHV-5), is a ubiquitous pathogen belonging to the β-herpesviridae subfamily of the *Cytomegalovirus* genus. HCMV infections usually show minor, nonspecific clinical symptoms in healthy individuals and can establish life-long latency in hosts [1]. Yet, primary HCMV infection or reactivation can cause high morbidity and mortality in immunocompromised patients such as those with immunosuppression after organ or bone marrow transplantation [2]. Additionally, the virus is frequently linked to congenital infections. The incidence of congenital HCMV infection ranges from 0.3~1.2% worldwide [3]. Among congenitally infected infants, about 7–10% will suffer from disabilities including mental retardation, loss of hearing or sight, microcephaly, and psychomotor dysfunction [1,4]. Horizontal HCMV transmission happens through close contact, while maternal-to-fetal transmission occurs when women contract HCMV during pregnancy [1]. Despite close to five decades of effort, no vaccine against HCMV has been licensed. Adverse effects and concerns of viral resistance limit the clinical use of antiviral drugs such as ganciclovir and valganciclovir [5]. Thus, an effective preventive vaccine and new potent drugs against HCMV infection are urgently needed. Understanding of viral entry mechanism may provide a new scientific basis for design of novel drugs or vaccines.

HCMV possesses a large (~235 kb) double stranded DNA genome packaged inside an icosahedral capsid with a diameter of 100–110 nm [1]. A protein layer known as tegument lies between the genome containing capsid and glycoprotein-embedded viral envelope membrane [1,6]. The HCMV genome is estimated to be capable of encoding more than 160 proteins. About 70 viral proteins are incorporated into the mature viral particle [7,8]. The complexity of the viral particle and long-term co-evolution with its host enable HCMV to infect a variety of human cell types. These cell types include epithelial cells, endothelial cells, fibroblasts, hepatocytes, neurons, parenchyma cells, and mononuclear cells [9].

For enveloped viruses such as HIV-1, influenza virus, and Ebola virus, a single viral glycoprotein is sufficient for cell attachment and membrane fusion that lead to successful infection of target cells [10]. In contrast, cell attachment and membrane fusion for herpesvirus including HCMV seem to be accomplished by different viral glycoprotein complexes. Together with gH and gL, glycoprotein B (gB), a highly conserved class III fusion protein among herpesvirus [11], forms the minimal fusogenic machinery necessary for viral-cellular membrane fusion [12]. For HCMV, two glycoprotein complexes, the gH/gL/gO (also termed trimer) and gH/gL/pUL128-131 (termed pentameric complex, or pentamer), co-exist on low-passage clinical viral strains. These complexes have been shown to play roles in modulation of viral cell tropism through interaction with different cellular receptors [13,14]. The trimer is sufficient for HCMV infection of fibroblast cells and the pentameric complex is specifically needed for HCMV infection of epithelial cells, endothelial cells, monocytes, and macrophages [15–17]. Laboratory-adapted HCMV strains such as AD169 gain null mutations at the UL131-128 locus during passage adaptation in fibroblast cells, resulting in failure of pentamer expression. These tissue culture-adapted viruses cannot infect epithelial and endothelial cells [18–21]. Restoration of pentamer expression by culture adaption or complementation can rescue viral tropism in epithelial and endothelial cells. The restoration can also be accomplished by repairing mutations in viral UL131-128 locus, as demonstrated for AD169 virus [18,21,22].

Multiple cellular molecules have been reported to be receptors or facilitators of HCMV infection *in vitro*. Heparan sulfate proteoglycans (HSPG) and β1-integrin are necessary for initiation of HCMV infection through interaction with gB [23–26]. Epidermal growth factor receptor (EGFR) activation by HCMV and its interaction with gB have been reported to play a role, potentially as a receptor, in HCMV infection [27–29]. However, other studies dispute the role of EGFR as a receptor necessary for HCMV infection [30,31]. The interaction of platelet-derived growth factor receptor-α (PDGFR-α) with gH/gL/gO trimer to facilitate HCMV infection in fibroblast cells has been confirmed in several studies [32–34]. Interestingly, knockdown of PDGFR-α only affects HCMV entry into fibroblast cells but not its entry into epithelial or endothelial cells [31]. THY-1 cell surface antigen (CD90) is reported to play a role in the early stage of HCMV infection, probably by facilitating viral entry via a macropinocytosis-like process [35]. CD147 has been reported to promote pentamer-expressing HCMV entry into epithelial and endothelial cells, but no direct interaction between CD147 and pentamer or trimer was verified [36]. Neuropilin-2 has been identified as a pentamer-binding receptor responsible for HCMV infection of epithelial and endothelial cells through screening a comprehensive membrane protein library [37]. A recent study identified OR14I1 as an additional pentamer-dependent host receptor associated with HCMV epithelial tropism [38]. Adding to the complexity of its infection strategy, HCMV may enter different cell types via different routes or mechanisms. For example, HCMV could enter fibroblast cells through direct fusion with the plasma membrane or macropinocytosis [35,39–42]. On the other hand, its entry into epithelial and endothelial cells may rely on endocytosis and low-pH [41,43,44]. With full appreciation of the long evolution of HCMV infection in a variety of human cell types, it is not surprising that more cellular factors are identified to play roles in HCMV entry process.

A previous study revealed that the laboratory-adapted HCMV strain is internalized into epithelial cells at the same rate as that of low-passage clinical strain. However, the laboratory-adapted strain was destined for degradation while the clinical strain somehow managed to accomplish membrane fusion in a low pH-dependent manner [31]. Recombinantly expressed soluble pentamer has been shown to inhibit HCMV infection of epithelial cells [45] and bind to the surface of epithelial cells [14]. In this study, 23 potential pentamer-binding membrane proteins were identified through pentamer pull-down. To validate their necessity for HCMV infection, these pentamer-binding candidates were individually knocked out in epithelia-derived ARPE-19 cells. One of the candidate proteins, adipocyte plasma membrane associated protein (APMAP), was found necessary for HCMV infection in epithelial cells and fibroblast cells. Over-expression of human APMAP promoted HCMV infection of two non-human cell types less susceptible to HCMV entry, canine MDCK and murine NIH/3T3 cells. Dramatically reduced immediate early gene (IE) transcription and delayed nuclear translocation of tegument protein pp65 were detected in APMAP-deficient cells at early stage HCMV infection. The *in vitro* interaction between APMAP and HCMV proteins by biochemical assays supported the notion that APMAP may participate in HCMV infection through interaction with gH/gL containing glycoprotein complexes at low pH and through mediating nuclear translocation of pp65. Taken together, our results indicated that APMAP is a cellular factor augmenting HCMV infection at the early stage of viral infection.

## Results

### Identification of APMAP as a cellular factor in HCMV infection

**Fig 1:**
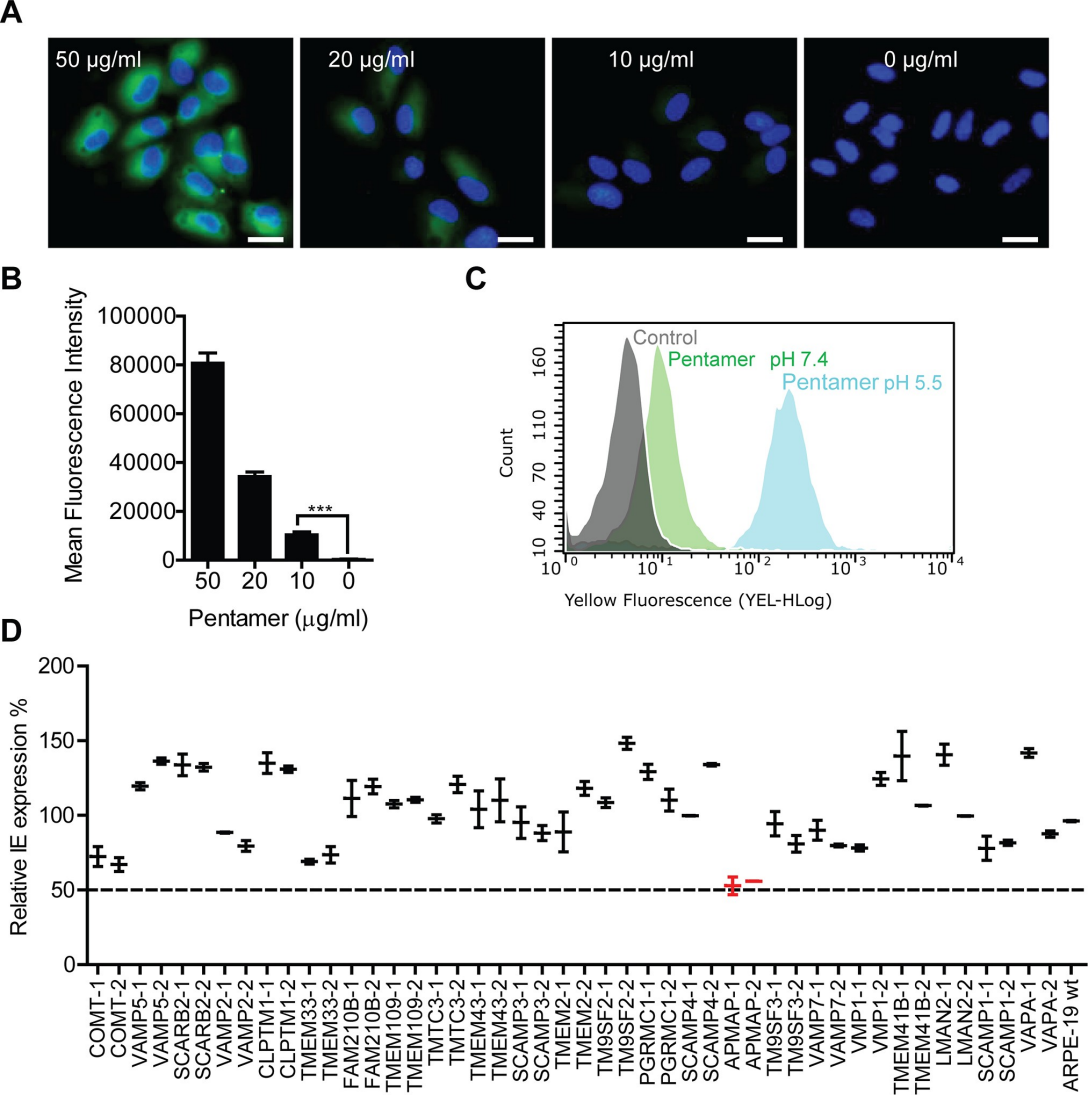
Interaction of HCMV pentamer with ARPE-19 cells. (**A**) AREP-19 cells grown in chamber slides were fixed by 4% PFA and blocked with 1% (W/V) BSA. Pentamer was diluted using 0.1% (W/V) BSA in PBS (pH7.4) at indicated concentrations and incubated with ARPE-19 cells for 1 h at 37°C. The cells were washed 3 times to remove unbound pentamer before staining with FITC-conjugated anti-His-tag antibodies which recognize recombinant gH subunit of the pentamer. Nucleus was stained with DraQ5. The cells were imaged using a LEIKA confocal microscopy. Bar = 20 nm. (**B**) Quantitation of pentamer specific fluorescence intensity in panel 1A. The unpaired two-tailed student t-test was used for significance analysis. (**C**) Pentamer was incubated with ARPE-19 cells in suspension at a final concentration of 20 μg/ml in either neutral pH (7.4) or low pH (5.5) buffer for 1 h on ice. After washing away of unbound pentamer, the cells were stained with FITC-conjugated anti-His tag antibodies and subjected to flow cytometry analysis. Cells incubated in buffer without pentamer but stained with the FITC-conjugated anti-His tag antibodies served as a negative control. (**D**) Top 23 candidate membrane proteins were knocked out (K/O) in ARPE-19 cells using the CRISPR/Cas9 system. These proteins were identified as candidates for pentamer binder at low pH by Pull-down/Mass spectrometry assay. The stable K/O cells were infected with HCMV strain AD169rev at MOI = 0.1 for 24 h and then subjected to IE protein staining by In-cell western assay. Data were shown as the percentage of IE signal in the K/O cells to that of infected wildtype ARPE-19 cells. The bars represent means ± SD of replicate wells. The data shown are representative results of three independent experiments.

Soluble pentamer can inhibit HCMV infection of ARPE-19 cells in a dose-dependent manner [45]. The binding of soluble pentamer, with 6×His-tag at C-terminus of the gH subunit [45], to the surface of ARPE-19 cells was determined. Soluble pentamer was incubated with ARPE-19 cells grown on chamber slides and stained with FITC-conjugated anti-His-tag antibodies. As shown in **Fig 1A and 1B**, the fluorescent signal for pentamer was weak at the lowest concentration (10 μg/ml) tested, and the signal increased in correlation with the pentamer concentrations, indicating dose-dependent binding of pentamer to ARPE-19 cells. Since pentamer-dependent HCMV infection of epithelial cells has been reported to rely on low pH [43], we compared pentamer binding on ARPE-19 cells at neutral pH (7.4) and low pH (5.5) buffer by flow cytometry assay. As shown in **Fig 1C**, pentamer binding in neutral pH buffer (green histogram) had a small but distinct shift in fluorescent intensity compared to that of a negative control (grey histogram), while pentamer binding in low pH buffer (blue histogram) had a more than one log of magnitude shift in fluorescent intensity as compared to the negative control. In contrast, no pentamer binding was detected on human MRC-5 fibroblast cells and non-human MDCK cells (**S1A and S1B Fig**). These results demonstrated that pentamer binds on ARPE-19 cells directly and the binding was enhanced by at least 10-fold at low pH.

To identify potential binding partners on ARPE-19 cells, a pentamer-specific monoclonal antibody 2–25 [46,47] was used to pull down extracted ARPE-19 total membrane proteins that were pre-incubated with soluble pentamer in low pH (5.5) buffer (**S1C Fig**). After mass spectrometry analysis of the pull-down sample, 23 candidates were chosen for further validation based on the criterion that they are membrane proteins with defined extracellular domains (**S1D Fig**). The CRISPR/Cas9 system was used to knockout these 23 targets individually in ARPE-19 cells. Then the knockout (K/O) stable cells were sub-cloned and two representative clones for each target were screened for infection by AD169rev, an AD169 revertant virus with pentamer expression restored, at multiplicity of infection (MOI) of 0.13 [48]. Virus infection in the cells was quantified by in-cell western assay through staining immediate early (IE) protein at 24 h after infection using a method as described [49]. Relative percentages of IE protein expression in the knockout cell lines to those of infected parental APRE-19 cells were calculated. As shown in **Fig 1D**, the stable clones with APMAP gene knockout showed the lowest levels of IE protein expression, correlated with the most pronounced effect on HCMV infection. Therefore, APMAP was chosen for further studies of its role in HCMV infection.

### Necessity of APMAP for HCMV infection of epithelial cells

**Fig 2:**
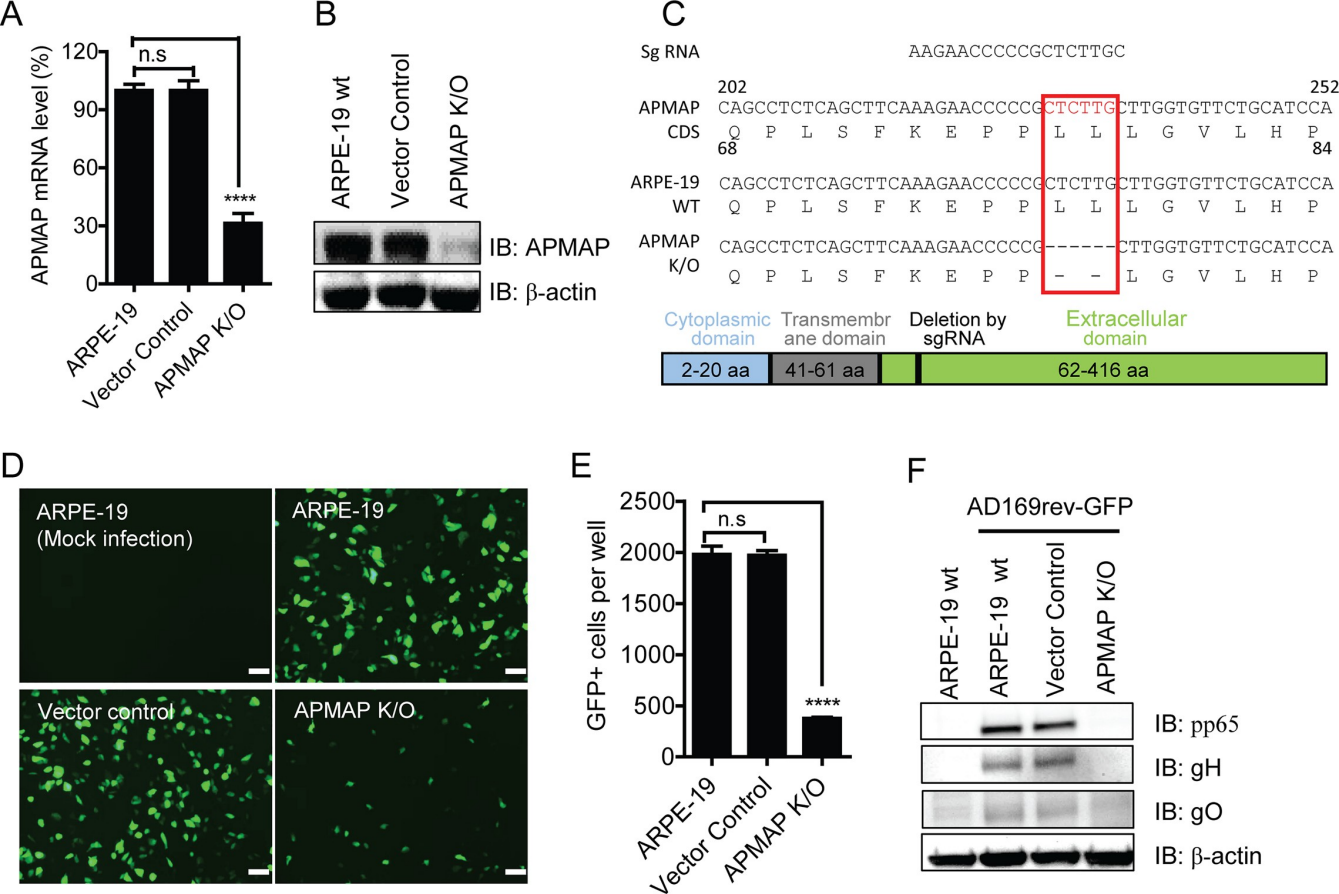
Reduced HCMV infection in APMAP K/O ARPE-19 cells. (**A**) APMAP mRNA level in wildtype ARPE-19, vector control and APMAP K/O cells were quantified by RT-qPCR. GAPDH mRNA served as internal control. The data were shown as relative APMAP mRNA level to that of wildtype ARPE-19 cells. The bars represent means ± SD of replicate wells. (**B**) APMAP knockout in ARPE-19 cells were confirmed by western blot analysis using 4F6 mAb which recognizes APMAP. GAPDH served as loading control. (**C**) Sequencing of APMAP gene of wildtype ARPE-19 and the APMAP K/O cells revealed two amino acids deletion in APMAP genes near the transmembrane domain in the K/O cells. (**D**) Wildtype ARPE-19 and APMAP K/O cells grown in 96-well plate were infected with AD169rev-GFP at MOI = 1.0, and the images were taken at 48 h after infection to detect GFP expression. Bar = 100 μm. (**E**) Quantitation of GFP positive cells in (D). Total number of GFP positive cells per well were shown. The bars represent means ± SD of GFP positive cells in four replicate wells. (**F**) Detection of HCMV protein expression in (D) by western blot analysis using anti-gH, anti-pp65, and anti-gO antibodies, respectively. β-actin served as loading control. The APMAP mRNA level (%) or number of GFP+ cells in vector control and APMAP K/O cells were compared individually to that of wildtype ARPE-19 cells using unpaired two-tailed student t-test for significance analysis.

The APMAP knockout (K/O) ARPE-19 cells used in initial screening were characterized by quantitation of APMAP mRNA using qRT-PCR and detection of APMAP protein by western blot assay with APMAP-specific antibody, respectively. As shown in **Fig 2A and 2B,** APMAP mRNA and protein levels in the K/O cells were significantly lower than those in ARPE-19 cells or in vector control ARPE-19 cells with scrambled sgRNA which does not target any gene. Sequencing analysis revealed that 6 nucleotides around the sgRNA targeting site were deleted. These deletions resulted in loss of 2 amino acids near the transmembrane domain of APMAP (**Fig 2C**). APMAP gene was not knocked out in APMAP K/O ARPE-19 cells per se, but APMAP expression in the cells was substantially reduced. To better track further results in this APMAP deficient cell line, the name APMAP K/O ARPE-19 was continue used for this cell line. Next, we sought to confirm the effect of reduced expression of APMAP on HCMV infection. APMAP K/O cells were infected with AD169rev-GFP (MOI = 1.0), an AD169rev virus with a GFP expression cassette [50]. Its counterpart is AD169-GFP, which has no pentamer expression. As expected, AD169-GFP and AD169rev-GFP grew in MRC-5 fibroblast cells with similar kinetics (**S2A Fig**). However, only AD169rev-GFP with restored pentamer expression grew in ARPE-19 cells (**S2A Fig**). GFP expression driven by SV40 early promoter in AD169rev-GFP or AD169-GFP infected cells enabled visualization of successful viral entry. As shown in **Fig 2D and 2E**, at 48 h after infection with AD169rev-GFP, the number of GFP positive cells among infected APMAP K/O cells was ~ 70% lower than the number of GFP positive cells among infected ARPE-19 cells or vector control cells. Accordingly, the expression levels of HCMV proteins pp65, gH, and gO were lower in infected APMAP K/O cells compared to that in infected wildtype and vector-control cells at 72 h after infection (**Fig 2F**). To evaluate the effect of MOI, the cells were infected with AD169rev-GFP at different MOIs (0.2, 1.0 and 5.0). APMAP K/O cells had lower relative percentage of GFP positive cells than did ARPE-19 or vector control cells at each tested MOI (**S2B Fig**). This result suggests that inhibition of virus infection in APMAP K/O cells is not affected by MOI. Taken together, these results demonstrate that HCMV infection is inhibited in ARPE-19 cells with deficient APMAP expression.

The effect of APMAP deficiency on HCMV entry in other epithelial cell lines were determined. HepG2 cells are derived from human hepatocellular carcinoma with human epithelial cell characteristics. HepG2 cells support HCMV entry but not productive viral replication [51]. A set of five short hairpin RNA (shRNA) constructs targeting APMAP were delivered into HepG2 cells through lentiviral vectors to knockdown (K/D) APMAP expression. As shown in **S3A Fig**, APMAP protein expression in stable cells with APMAP-specific shRNA, but not in the control cells with scramble shRNA (sc-shRNA), was lower than in wildtype HepG2 cells. At 48 h after infection with AD169rev-GFP, the number of GFP positive cells among the APMAP K/D cells were significantly lower (up to 70%) than that in wildtype HepG2 cells and cells with sc-shRNA. This pattern was observed regardless of testing MOI at 10, 2.0 or 0.4 (**S3B and S3C Fig**). RT-qPCR analysis of infected cells (MOI = 2.0) revealed that viral IE mRNA levels were also significantly lower in APMAP K/D cells than in wildtype HepG2 cells (**S3D Fig**). In addition, we tested the effect of APMAP knockdown in Hela cells. As expected, APMAP protein expression were decreased in the stable cells with APMAP-specific shRNA (**S4A Fig**). The infectivity of AD169rev-GFP as indicated by the number of GFP positive cells and IE mRNA levels at 48 h after virus infection (MOI = 1.0) were also significantly decreased in APMAP K/D cells than in wildtype Hela cells and Hela cells with sc-shRNA (**S4B–S4D Fig**). Taken together, these results indicated that APMAP is necessary for HCMV infection of human epithelial cells.

### Necessity of APMAP for HCMV infection of fibroblast cells

**Fig 3:**
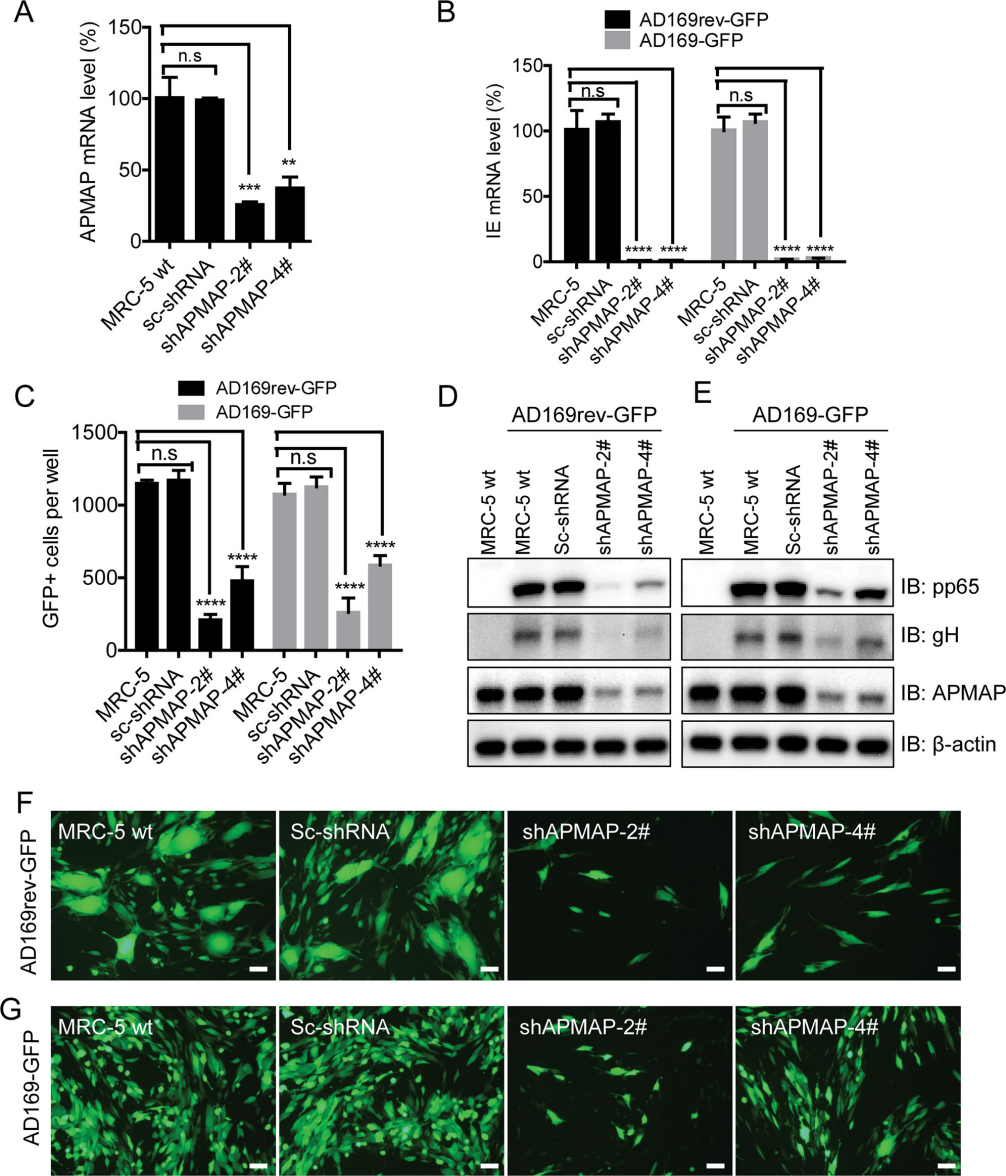
Reduction of HCMV infection in APMAP K/D MRC-5 cells. (**A**) APMAP knockdown MRC-5 stable cells were generated by infection with lentivirus expressing APMAP specific shRNA under puromycin selection. APMAP mRNA level in the knockdown cells were detected by RT-qPCR. GAPDH mRNA served as internal control. The data are shown as percentages of APMAP mRNA levels in knockdown cells relative to that of MRC-5 cells. (**B**) Wildtype and APMAP K/D MRC-5 cells were infected with AD169rev-GFP and AD169-GFP at MOI = 1.0 in 96-well plate, respectively. 6 h after infection, the cells were collected for RNA extraction and RT-qPCR to detect viral IE mRNA level. The data were shown as percentages of IE mRNA level in infected knockdown cells relative to that of MRC-5 cells. GAPDH mRNA served as internal control. (**C-G**) In another HCMV infection experiment as in Fig 4B, (**C**) 48 h after infection, the plate was read by C.T.L. Immunospot machine to capture images under fluorescence cell mode for GFP. GFP positive cells in each well were counted automatically using the software. The data are shown as means ± SD of total number of GFP positive cells per well for four replicate wells. (**D-G**) 72 h after infection, (**F-G**) representative images (Bar = 100 μm) showing overall GFP positive cells were captured by Olympus fluorescence microscopy; and then (**D-E**) the cells were collected for western blot assay to detect HCMV protein expression using anti-pp65, anti-gH and anti-APMAP (4F6) antibodies, respectively. β-actin served as loading control. The APMAP mRNA level (%), IE mRNA level (%), or number of GFP+ cells in sc-shRNA or shAPMAP expressing cells were compared individually to that of wildtype MRC-5 cells using unpaired two-tailed student t-test for significance analysis. The data shown are representative results of two independent experiments.

MRC-5 is a human fibroblast cell line susceptible to HCMV infection, regardless of the pentamer expression. To determine whether APMAP is necessary for HCMV infection of fibroblast cells, stable APMAP K/D MRC-5 cells were generated as described above. Levels of APMAP mRNA were much lower in APMAP K/D MRC-5 cells than in MRC-5 cells or in sc-shRNA control cells **(Fig 3A)**. We next sought to determine the function of APMAP in pentamer-associated viral infection. AD169-GFP and AD169rev-GFP were used to infect APMAP K/D MRC-5 cells at a MOI of 1.0. At 6 h post infection, viral IE mRNA levels were more than 95% lower in APMAP K/D cells infected with either AD169-GFP or AD169rev-GFP than in control cells (**Fig 3B**). At 48 h post infection, the numbers of GFP positive cells among AD169-GFP and AD169rev-GFP infected APMAP K/D cells were significantly lower than that of infected sc-shRNA control and wildtype MRC-5 cells (**Fig 3C**). Notably, the reduction in AD169-GFP infection versus AD169rev-GFP infection in APMAP K/D cells were comparable. At 72 h post infection, viral protein expression in the cells was assessed by western blot analysis. The expression levels of viral proteins pp65 and gH were dramatically lower in APMAP K/D cells than in wildtype and sc-shRNA control cells. These lower expression levels were in parallel to the lower expression level of APMAP protein (**Fig 3D and 3E**). Lower viral protein expression levels were consistent with lower levels of corresponding viral mRNA transcription as assessed by qRT-PCR at 72 h after infection (**S5 Fig**). The number of cells with GFP expression at 72 h post infection was also significantly lower in APMAP K/D cells than that of control cells (**Fig 3F and 3G**) for both AD169-GFP and AD169rev-GFP infection. These results indicate that APMAP is necessary for HCMV infection of MRC-5 fibroblast cells and potentially acts before transcription of IE gene. The entry of both AD169-GFP and AD169rev-GFP were affected in APMAP K/D MRC-5 cells, suggesting that APMAP is not involved in HCMV infection in a pentamer specific manner.

### Promotion of HCMV entry by over-expression of APMAP

**Fig 4:**
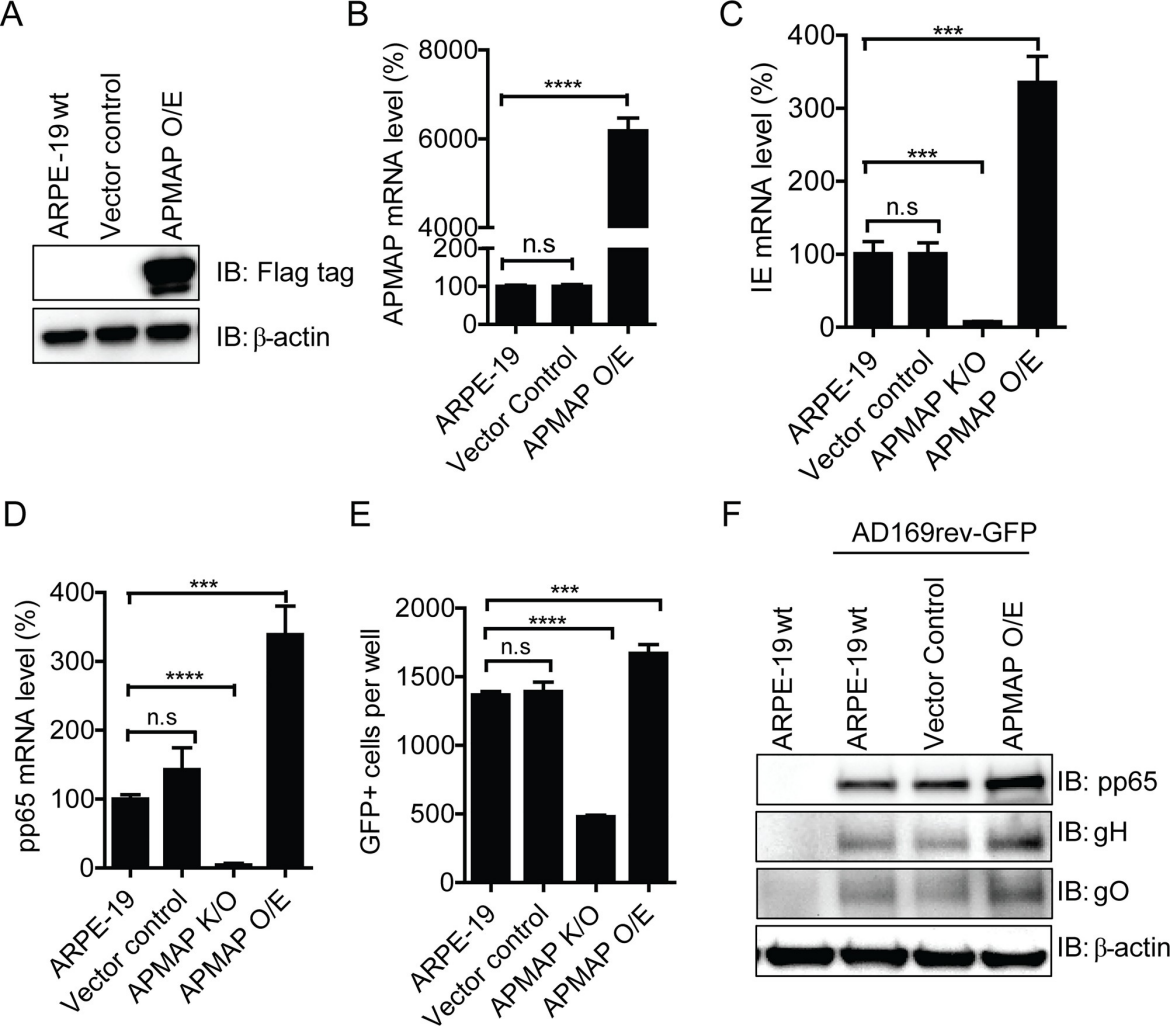
Enhanced AD169rev-GFP infection of ARPE-19 cells over expressing APMAP. (**A-B**) APMAP O/E stable cells were established by infecting ARPE-19 cells with lentivirus particles capable of expressing full length APMAP coding sequence with a Myc/Flag tag at C-terminus under puromycin selection. APMAP over expression in ARPE-19 cells were confirmed by (**A**) western blot using mouse mAb anti-Flag tag and (**B**) RT-qPCR, respectively. GAPDH served as internal control. (**C-E**) ARPE-19 APMAP K/O, APMAP O/E, and control cells were infected with AD169rev-GFP at MOI = 1.0 in 96-well plate. At 8 h after infection, the cells were collected for RNA extraction and RT-PCR to detect (**C**) HCMV IE mRNA level and (**D**) pp65 mRNA level, respectively, and shown as percentages to that infected ARPE-19 cells, with GAPDH mRNA served as internal control. No significant signal for IE and pp65 mRNA in mock-infected cells could be detected by the primers and thus was not shown. Data analysis was performed using the 2^-ΔΔCT^ method. The data are shown as relative IE or pp65 mRNA level to that of infected wildtype cells. The black bars represent means ± SD of replicate wells. (**E**) In another infection experiment, the plate was read by C.T.L. Immunospot machine at 48 h post infection. Number of GFP positive cells were counted automatically using the software. The data are shown as means ± SD of total number of GFP positive cells per well for triplicate wells. The APMAP mRNA level (%), IE mRNA level (%), or number of GFP+ cells in vector contol, APMAP K/O and APMAP O/E cells were compared individually to that of wildtype MRC-5 cells using unpaired two-tailed student t-test for significance analysis. (**F**) At 72 h after infection, the cells were collected to detect HCMV protein expression by western blot, using anti-pp65, anti-gH and anti-gO antibodies, respectively. β-actin served as loading control.

The current data suggest that APMAP was not one of the cellular receptors that interact specifically with the pentamer for viral entry. However, results indicate a significant role for APMAP in viral entry at the early stage of viral infection for the cell types tested. To better understand the role of APMAP in viral entry, we over expressed human APMAP with a C-terminal Myc/Flag-tag in ARPE-19 cells. Protein expression in ARPE-19 stable cells overexpressing (O/E) APMAP was confirmed by western blotting using a Flag-tag specific antibody (**Fig 4A**). Further, a more than 60-fold increase in APMAP mRNA was measured by qRT-PCR (**Fig 4B**). At 8 h after infection by AD169rev-GFP (MOI = 1.0), relative mRNA levels of viral IE and pp65 in APMAP O/E cells, as determined by qRT-PCR, were ~2-fold higher than that in vector control cells (**Fig 4C and 4D**). In contrast, relative viral IE and pp65 mRNA levels in the APMAP K/O cells, which were included as control, were ~ 90% lower than that of infected wildtype or vector control cells (**Fig 4C and 4D**). The number of GFP positive cells was also modestly higher in APMAP O/E ARPE-19 cells (**Fig 4E**). Consistent with higher viral mRNA detected at 8 h post infection in APMAP O/E ARPE-19 cells, the western blot assay also showed that expression of viral proteins pp65, gH, and gO in APMAP O/E ARPE-19 cells at 72 h post infection were slightly higher than those in the wildtype and control cells (**Fig 4F**). Without pentamer expression, AD169 strain infects ARPE-19 cells poorly. To determine whether over-expression of APMAP in ARPE-19 cells can bypass the requirement for pentamer expression, AD169-GFP was used to infect APMAP O/E cells (MOI = 1.0). GFP expression in the cells was recorded at 72 h post infection. As expected, only a few GFP positive cells were captured in each image (**S6A Fig**) and the total numbers of GFP positive cells per well were also a lot less than that of infected by AD169rev-GFP at same MOI (**Fig 4E** and **S6B Fig**). The number of GFP positive cells was higher among infected APMAP O/E ARPE-19 cells and significantly lower in infected APMAP K/O cells than in infected ARPE-19 cells (**S6B Fig**). The levels of viral IE mRNA and pp65 mRNA were also significantly higher in infected APMAP O/E cells and lower in infected APMAP K/O cells than in infected ARPE-19 cells (**S6C Fig**). By western blot analysis, viral protein pp65 and gH were clearly detected in APMAP O/E cells but barely detectable in control cells at 6 days after infection (**S6D Fig**). Collectively, these results show that over expression of APMAP in ARPE-19 cells slightly enhanced infection by HCMV strains with and without pentamer, which led to enhanced transcription of viral genes and expression of viral proteins.

**Fig 5:**
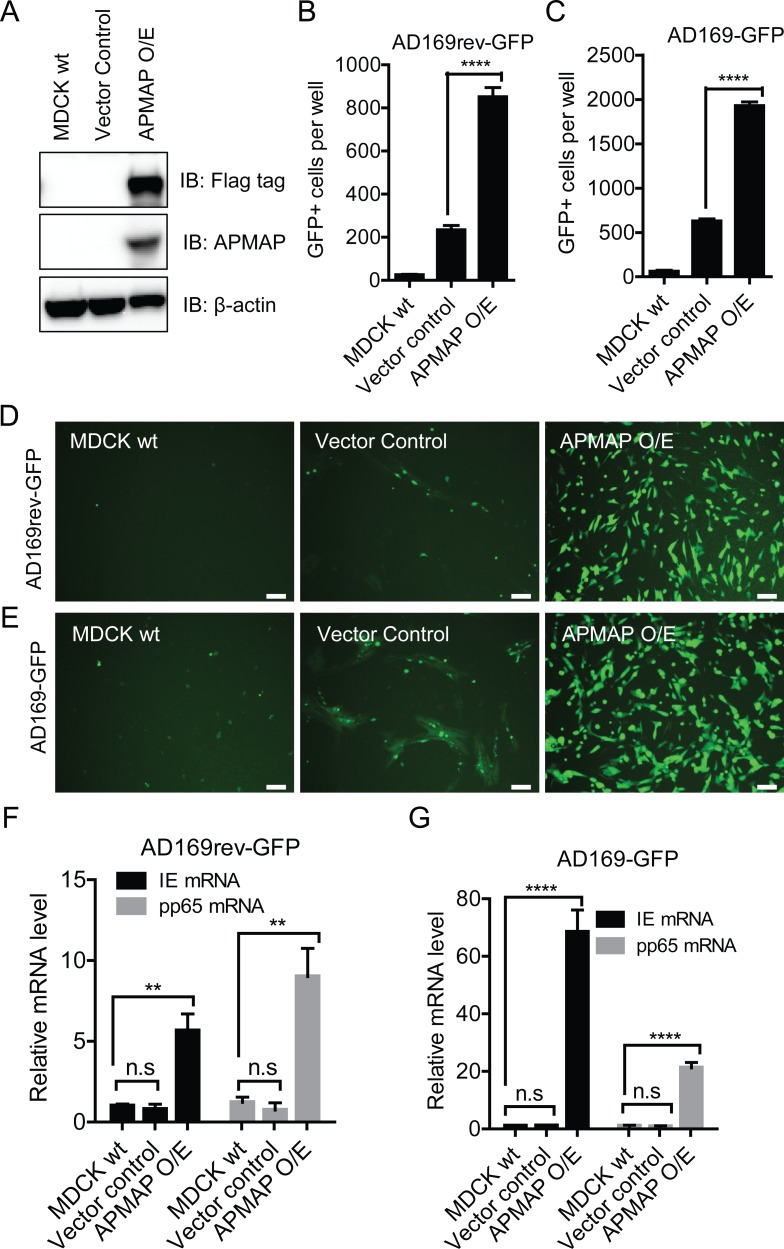
Expression of human APMAP permitted HCMV entry in MDCK cells. (**A**) APMAP O/E stable cells were established by infecting MDCK cells with lentivirus particles capable of expressing human full length APMAP coding sequence with a myc/flag tag at C-terminal under puromycin selection. APMAP over expression in MDCK cells were confirmed by western blot analysis using mouse anti-Flag tag or anti-APMAP (4F6) antibodies. β-actin served as loading control. (**B-G**) Wildtype MDCK and APMAP O/E stable cells were infected with AD169rev-GFP and AD169-GFP at MOI = 1.0 in 96-well plate, respectively. (**B-C**) At 2 days post infection, the plate was read by C.T.L. Immunospot machine to capture images under fluorescence cell mode for GFP. GFP positive cells in each well were counted automatically using the software. The data are shown as means ± SD of total number of GFP positive cells per well for four replicate wells. (**D-E**) Representative images (Bar = 100 μm) showing overall GFP positive cells at day 2 post infection by (**D**) AD169rev-GFP or (**E**) AD169-GFP were captured using an Olympus fluorescence microscope. (**F-G**) The cells were collected at 2 days after infection for qRT-PCR detection of viral IE and pp65 mRNA. GAPDH mRNA served as internal control. Data analysis was performed using the 2^-ΔΔCT^ method. The data are shown as relative percentages of IE or pp65 mRNA level to that of infected wildtype cells. The number of GFP+ cells, relative IE mRNA level or pp65 mRNA level in vector contol and APMAP O/E cells were compared individually to that of wildtype MDCK cells using unpaired two-tailed student t-test for significance analysis. The black bars represent means ± SD for triplicate wells.

Two non-human cell lines were selected to test whether human APMAP can enhance HCMV infection. MDCK, a dog kidney-derived epithelial cell line, was modified for overexpression of full length human APMAP with a C-terminal Myc/Flag-tag. Over expression of APMAP protein was confirmed by blotting with both APMAP and the flag tag specific antibodies (**Fig 5A**). Interestingly, the number of GFP positive cells significantly increased in APMAP O/E cells than in control cells at 48 h post infection by either AD169-GFP or AD169rev-GFP (MOI = 1.0) **(Fig 5B and 5C)**. Under fluorescent microscopy, wildtype MDCK cells or those with vector control exhibited very low levels of HCMV infection as indicated by GFP expression, while the APMAP O/E MDCK cells showed significantly more HCMV infection as indicated by GFP expression (**Fig 5D and 5E**). Consistently, viral IE mRNA and pp65 mRNA levels were also significantly higher in APMAP O/E MDCK cells than in wildtype and vector control cells after infection by AD169-GFP and AD169rev-GFP (**Fig 5F and 5G**). In addition, human APMAP was overexpressed in the mouse fibroblast cell line NIH/3T3, which is not susceptible to HCMV infection (**S7A Fig**). Similarly, the infectivity of both AD169-GFP and AD169rev-GFP were significantly increased in human APMAP O/E NIH/3T3 cells than in control cells as indicated by GFP expression (**S7B–S7E Fig**) and viral IE/pp65 mRNA levels (**S7F and S7G Fig**). Further experiments showed that viral protein pp65 were expressed at higher levels in both infected APMAP O/E MDCK and APMAP O/E NIH/3T3 cells than in infected wildtype and vector control cells (**S8A and S8B Fig**). The infected APMAP O/E MDCK cells or APMAP O/E NIH/3T3 cells also produced more infectious virions than corresponding infection in wildtype and vector control cells (**S8C–S8F Fig**). These results demonstrate that human APMAP can improve HCMV infection in non-susceptible cells in a pentamer non-specific manner, consistent with its impact in ARPE-19 and MRC-5 cells.

Characterization of cell-growth rates and endocytosis function of the APMAP modified ARPE-19, MRC-5, Hela, HepG2, MDCK, and NIH/3T3 cell lines revealed that APMAP K/O, K/D, and O/E had negligible impact on global health of the modified cells (**S9 and S10 Figs**). Meanwhile, infection of the APMAP modified cells with herpesvirus simplex virus 2 (HSV-2) showed that deficiency in or overexpression of APMAP did not consistently affect HSV-2 infection in the tested cell lines (**S11 Fig**). These results suggest that effects of APMAP expression on HCMV infection were unlikely due to a common non-specific cellular function.

### Direct interaction of APMAP with HCMV structural proteins

**Fig 6:**
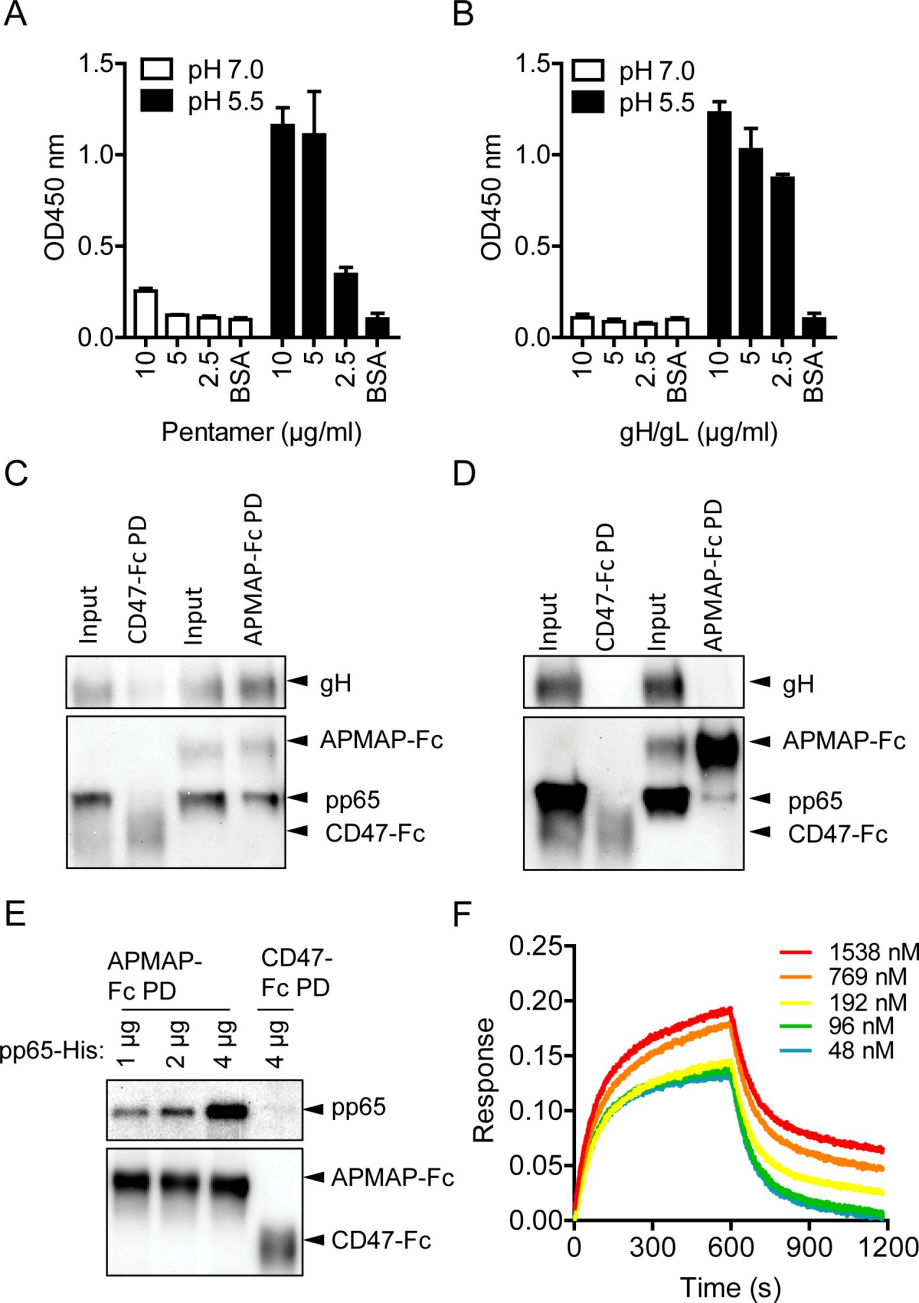
Interactions of APMAP with HCMV proteins. (**A-B**) The interaction of (**A**) Pentamer or (**B**) gH/gL dimer to APMAP at indicated concentrations in neutral pH 7.0 or acid pH 5.5 buffer was evaluated by ELISA assay. APMAP-Fc served as coating antigen. Pentamer was detected with HRP conjugated anti-His-tag antibodies which recognizes gH subunit of the pentamer. Samples with OD450nm readings that are 1.5 times higher than that of BSA control were considered positive. The data shown are representative results of two independent experiments. The bars represent means ± SD of replicate wells. (**C-D**) 1 μg APMAP-Fc or control CD47-Fc protein was incubated with 2.58×10^6^ PFU of AD169rev (**C**) in 500 μl PBS or (**D**) 500 μl RIPA buffer for 2 h at 4°C. The protein complex was pulled down by Protein G beads and analyzed by western blot assay using anti-gH mAb, anti-pp65 mAb and HRP conjugated anti-mouse IgG antibodies. (**E**) 2 μg APMAP-Fc or control CD47-Fc protein was loaded onto Protein G beads and then incubated with different amounts of His-tagged pp65 protein at room temperature for 1 h. After washing away of unbound protein, the beads were suspended into SDS containing loading buffer for western blot assay using mouse anti-His-tag mAb and HRP conjugated goat anti-mouse IgG secondary antibodies. (**F**) The binding of His-tagged pp65 to APMAP-Fc was detected by BLI assay using protein A sensors. The binding of kinetic buffer to APMAP-Fc loaded sensor were used as reference and subtracted before data analysis.

APMAP was identified by pull-down with soluble pentamer at low pH (**S1C Fig**). To determine whether there is a direct interaction between APMAP and soluble pentamer, the extracellular domain (62–416 aa) of APMAP with a C-terminal Fc-tag (APMAP-Fc) was generated (**S12A Fig**). Production of soluble gH/gL dimer, which has a 6×His-tag at the C-terminal of gH subunit, has been described previously [45]. The binding of soluble pentamer or gH/gL dimer to APMAP-Fc was assessed by bio-layer interferometry (BLI) assay. In neutral pH based kinetic buffer, a dose-related low binding of pentamer but not gH/gL to APMAP was detected (**S12B and S12C Fig**). By ELISA with APMAP-Fc as capture antigen, positive binding of pentamer was only detected at the highest concentration tested at neutral pH (7.4) and no positive binding of gH/gL dimer was detected at all concentrations tested at neutral pH (7.4) (**Fig 6A and 6B**), consistent with the results of BLI assay. Meanwhile, significantly increased and positive binding of both pentamer and gH/gL dimer were detected for all concentrations tested at low pH (5.5) (**Fig 6A and 6B**). In addition, pre-incubation with anti-gL and anti-APMAP polyclonal antibodies but not control antibodies inhibited the binding of pentamer to APMAP-Fc at low pH (**S12D and S12E Fig**). These results confirmed the interaction of APMAP with pentamer in low pH buffer and suggested that APMAP could bind pentamer through gH/gL subunit. These results partially explained the impact of APMAP on infection of AD169-GFP, which has gH/gL containing glycoprotein complexes other than pentamer.

Next, we sought to determine whether APMAP can interact with HCMV virions via the gH/gL glycoprotein complex. Purified HCMV virus [52] was incubated with APMAP-Fc or a control protein CD47-Fc in either PBS buffer or radio immunoprecipitation assay (RIPA) buffer. After pull-down with protein G beads, the samples were analyzed by western blot. As shown in **Fig 6C**, when HCMV virus was diluted in PBS, viral gH and pp65 were both detected in APMAP-Fc but not in CD47-Fc pull-down samples. Major tegument protein pp65 of intact virions should have no access to APMAP-Fc. This result suggests pull-down of whole virions by APMAP-Fc through its potential interaction with gH containing glycoproteins on virus surface. As shown in **Fig 6D**, when HCMV virus was diluted in RIPA buffer, which contains high concentration of detergents that can solubilize viral membrane proteins and eliminate weak protein-protein interactions, tegument protein pp65 would be exposed to APMAP-Fc. In this case, viral protein pp65 but not gH was detected in the APMAP-Fc pull-down sample; no viral protein could be detected in the CD47-Fc pull-down sample as expected. This result suggests a possible interaction between APMAP and pp65. Furthermore, recombinant expressed full-length pp65 with 6×His tag was pulled down by APMAP-Fc in a dose dependent manner (**Fig 6E**). The pp65 also showed a dose-related binding to APMAP-Fc by BLI assay (**Fig 6F**). These results further support the possible interaction between pp65 and APMAP.

### APMAP deficiency not affecting HCMV attachment and membrane fusion

**Fig 7:**
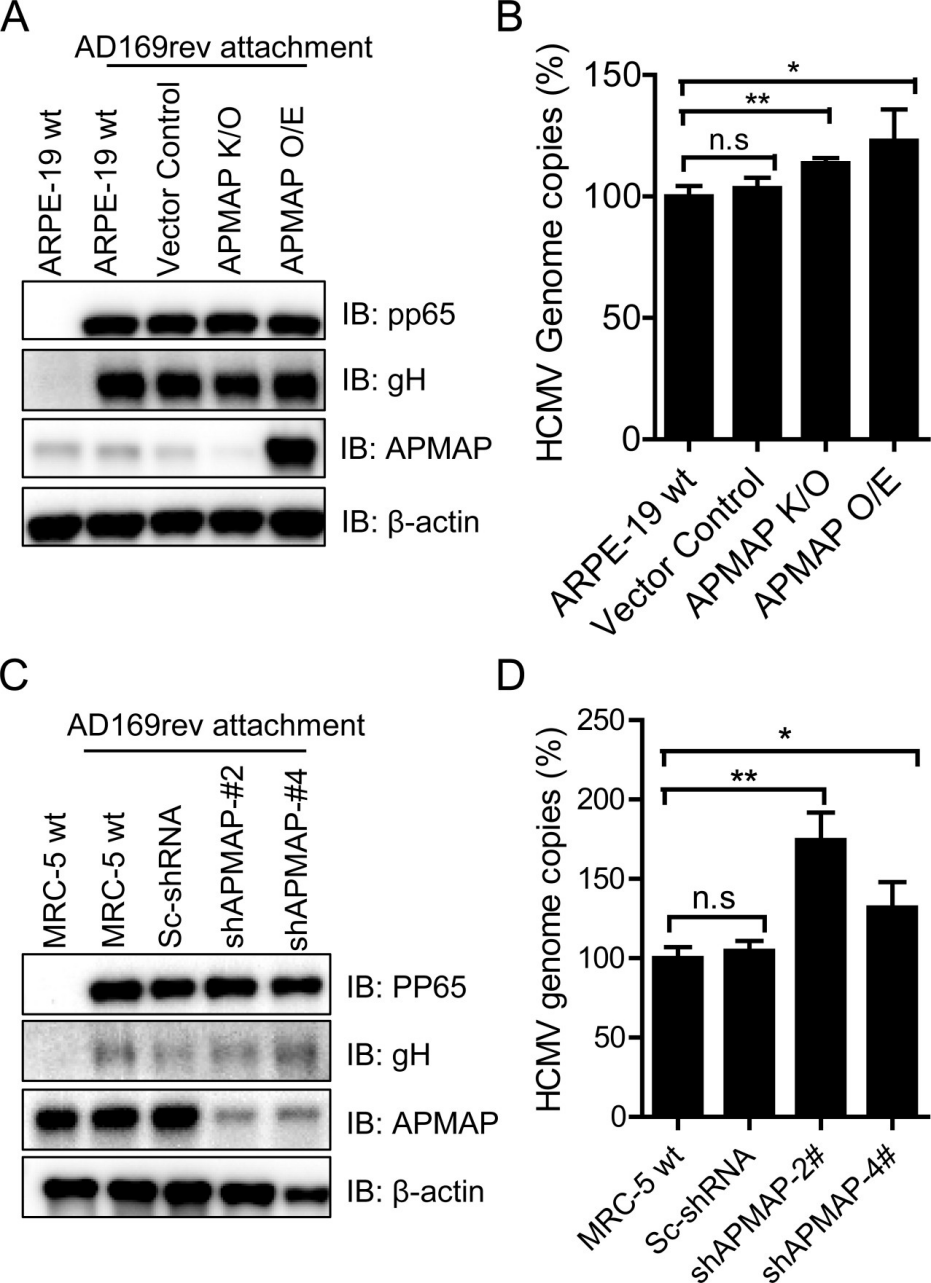
HCMV attachment was not affected in APMAP deficient target cells. (**A-B**) Wildtypte ARPE-19, APMAP K/O and APMAP O/E cells were seeded in 24-well plate (1×10^5^ cells/well) for 1 day. The cells were pre-cooled at 4°C for 10 mins. After removing the medium, 2.4×10^6^ PFU/well of AD169rev diluted in 200 μl complete medium was added to the cells and incubated at 4°C for 90 mins. After washing of unbound virus with cold PBS for 3 times, the cells were collected to determine HCMV attachment either by (**A**) western blot assay to detect viral proteins pp65 and gH, and APMAP with β-actin as loading controls; or by (**B**) qPCR using pp65 specific primers to quantify HCMV genome. Cells genomic DNA and HCMV genome were extracted together using Qiagen kit. The DNA samples were quantified by Nanodrop and same amount of DNA were used as template in qPCR. A plasmid inserted with pp65 genes, pET28a-pp65, was used generated standard curve. The data are shown as percentages of HCMV genomes on cells surface relative to that of wildtype ARPE-19 cells. Relative percentages of HCMV genome copies in vector control, APMAP K/O and APMAP O/E cells were compared individually to that of wildtype ARPE-19 cells using unpaired two-tailed student t-test for significance analysis. (**C-D**) AD169rev attachment on MRC-5 and APMAP K/D cells were determined as described above by (**C**) western blot and (**D**) qPCR assay, respectively, as described above.

Our data have shown that knockdown APMAP inhibits HCMV infection and overexpression of APMAP enhances HCMV infection (**Figs 2–5**). Further, we have shown that APMAP interacts with viral gH/gL containing glycoprotein complexes (**Fig 6**). Taken together, these results indicate that APMAP may play a role in HCMV attachment to host cells or membrane fusion with cell membrane. To test this hypothesis, we compared the attachment of AD169rev to APMAP K/O, APMAP O/E, Vector control and wild type ARPE-19 cells at 4°C. As shown in **Fig 7A**, western blot analysis of pp65 and gH proteins in virus attached cells revealed no distinct changes in the levels of viral proteins among these cells with wide range of APMAP expression levels. In addition, qPCR analysis of the HCMV viral genome after attachment showed less than 10% change in viral genome copy numbers between APMAP K/O cells and APMAP O/E cells (**Fig 7B**). Similar experiments were performed on MRC-5 cells. No reduction of HCMV viral attachment with APMAP K/D was measured by either western blot assay or qPCR (**Fig 7C and 7D**). These results collectively indicate that APMAP was not directly involved in viral attachment, and lower HCMV infection in APMAP deficient cells was not due to reduced HCMV attachment. Although APMAP interacts with gH/gL containing glycoprotein complexes, the interaction has negligible effect on HCMV attachment to ARPE-19 or MRC-5 cells. In addition, pre-incubation of soluble APMAP-Fc with AD169rev-GFP exhibited marginally inhibiting effect on virus infection in ARPE-19 cells (**S13A Fig**). This result further supports the conclusion that APMAP has no significant role in HCMV attachment.

Next, we sought to determine whether viral membrane fusion with target cells would be inhibited in cells with compromised APMAP expression. We performed a polyethylene glycol treatment assay. Wildtype and APMAP K/D MRC-5 cells were infected with AD169-GFP or AD169rev-GFP (MOI = 1.0) for 1 h, then treated with an artificial membrane fusion inducing agent polyethylene glycol 8000 (PEG-8K), which is widely used to study HCMV membrane fusion with target cells [43,53]. If viral membrane fusion was inhibited in the APMAP K/D cells, PEG treatment should be able to rescue viral infection in the K/D cells. As shown in **S13B and S13C Fig**, no significant difference of the numbers of GFP positive cells were observed between APMAP K/D cells with and without PEG-treatment for infection by either AD169-GFP or AD169rev-GFP. This result suggests that viral membrane fusion with target cells were not affected in APMAP K/D MRC-5 cells.

### Delayed nucleus translocation of pp65 in APMAP deficient cells

**Fig 8:**
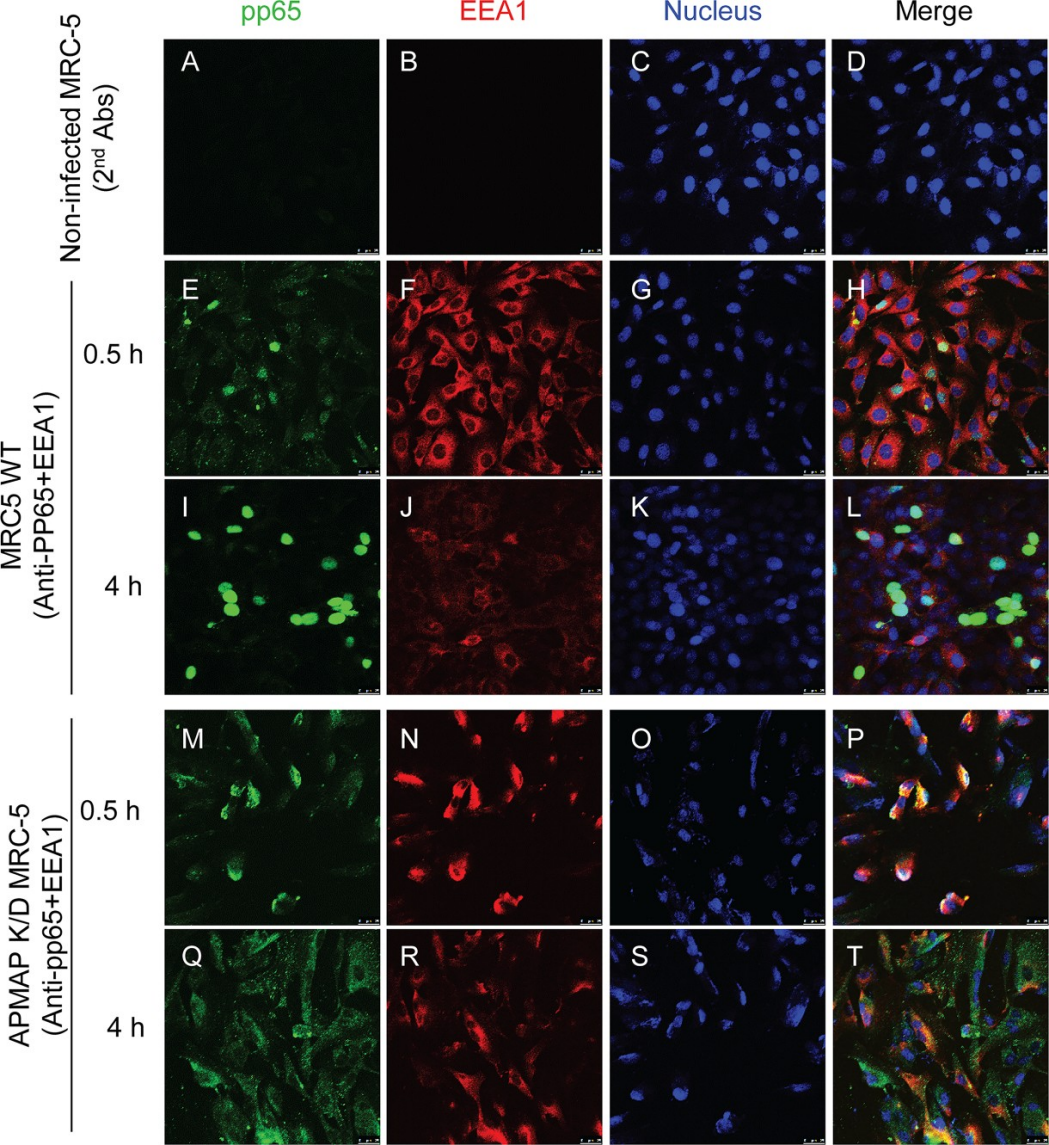
Delayed pp65 nucleus translocation in APMAP K/D MRC-5 cells. (**A-L**) Wildtype MRC-5 and (**M-T**) APMAP K/D cells were seeded in chamber slides (1.5×10^4^ cells/well) for 1 day. The cells were pre-cooled at 4°C before adding AD169rev for attachment at 4°C for 1 h to synchronize virus infection. Then, the cells were transferred to 37°C and cultured for 0.5 h or 4 h before fixation with 4% PFA. The cells were double stained with mouse anti-pp65 and Rabbit anti-EEA1 antibodies and corresponding Fluorescence labelled secondary antibodies. Nuclei were stained by DraQ5. The pictures were taken using LEIKA confocal microscopy. Bar = 25 μm.

**Fig 9:**
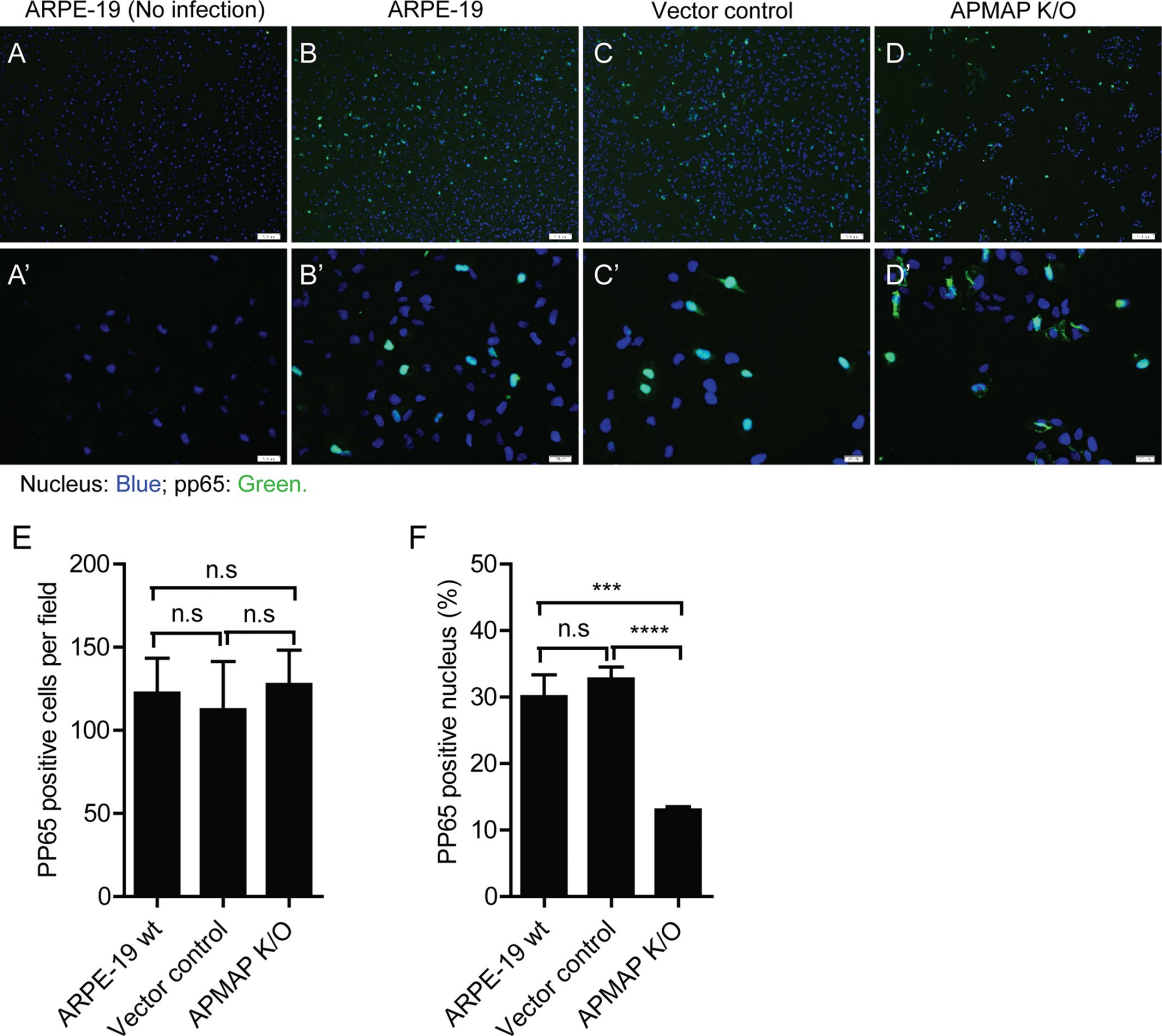
APMAP deficiency reduced pp65 translocation into nucleus. (**A-B, A’-B**’) Wildtype ARPE-19, (**C, C’**) vector control and (**D, D’**) APMAP K/O cells were seeded in chamber slides (1.5×10^4^ cells/well) for 1 day. The cells were pre-cooled at 4°C before adding AD169rev for attachment at 4°C for 1 h to synchronize virus infection. Then, the cells were transferred to 37°C and cultured for 3 h before fixation with 4% PFA. The cells were stained with mouse anti-pp65 antibodies and Alexa Fluo-488 conjugated anti-mouse IgG antibodies. Nuclei were stained by Hoechst. (A, A’) Mock-infected ARPE-19 cells were stained to serve as negative control. The images were captured using an Olympus fluorescence microscope. Panels A-D are representative images showing overall pp65 staining at lower magnification (Bar = 100 μm). Panels A’-D’ are representative images showing pp65 staining at higher magnification (Bar = 20 μm). (**E**) The number of pp65 positive cells were counted in four independent images for each sample as shown in panels A-D and plotted as Mean ± SD of GFP positive cells per field. (**F**) The number of pp65 positive nucleus in four independent images for each sample as shown in panels A-D were counted, respectively, and shown as percentages to the number of total pp65 positive cells in each image. Pairs of samples as indicated in the Fig 9E and 9F were compared individually using unpaired two-tailed student t-test for significance analysis. The data shown are representative results of two independent experiments.

To further understand the role of APMAP in HCMV infection, we monitored the internalization of HCMV at different times after infection. AD169rev was added to wildtype MRC-5 and APMAP K/D cells grown in chamber slides. To synchronize virus attachment, attachment was allowed to proceed at 4°C for 1 h. The cells were then immediately transferred for incubation at 37°C for 0.5 h or 4 h. Subsequently, cells were fixed and stained with pp65 specific antibody (green), early endosome antigen 1 (EEA1) specific antibodies (red), and nuclear stain DraQ5 (blue). Mock infected wildtype MRC-5 cells were stained and served as negative control (**Fig 8A–8D**). While pp65 were readily detected in both of wildtype and APMAP K/D MRC-5 cells at 0.5 h after infection (**Fig 8E–8H and 8M–8P)**, pp65 was primarily detected in the nucleus of the wildtype cells. This result was in contrast to those observed in APMAP K/D cells, where staining of pp65 occurred mostly in cytoplasm and co-localized with early endosome marker EEA1 (**Fig 8E–8H and 8M–8P**). Strong pp65 staining was detected almost exclusively in the nucleus of wildtype MRC-5 cells at 4 h after infection. On the other hand, relatively weak and scattered pp65 staining was detected in the cytoplasm of APMAP K/D cells, partially co-localized with EEA1 (**Fig 8I–8L and 8Q–8T**). Similarly, tegument delivered pp65 was also detected in wildtype and APMAP K/O ARPE-19 cells at 3 h post infection. The number of pp65 positive cells was comparable in wildtype or vector control ARPE-19 cells versus in APMAP K/O ARPE-19 cells (**Fig 9A–9D and 9E**). This result suggests that HCMV internalization is not affected in APMAP K/O ARPE-19 cells. Furthermore, pp65 was not efficiently translocated to the nucleus in most APMAP K/O ARPE-19 cells (**Fig 9A’–9D’**). The percentage of pp65 positive nucleus relative to total pp65 positive cells was significantly lower in APMAP K/O cells than in wildtype or vector control ARPE-19 cells (**Fig 9A’–9D’ and 9F**). These results indicate that nuclear translocation of tegument protein pp65 were delayed in APMAP deficient MRC-5 and ARPE-19 cells.

## Discussion

Herpesviruses use distinct cellular receptors and entry routes to infect different cell types. For example, Epstein-Bar virus (EBV) utilizes CD21, CD35, and HLA class II to enter B cells via endocytosis and pH-independent fusion with endosome membrane [54,55]. On the other hand, it utilizes ephrin receptor A2 (EphA2), integrins, and NMHCIIA to infect epithelial cells through direct fusion with the plasma membrane [56]. HSV-1 relies on one of the gD-interacting cellular receptors such as herpes virus entry mediator (HVEM), nectin-1, or 3-O-sulfated heparan sulfate to enter Vero and Hep2 cells via direct fusion at the plasma membrane. In contrast, entry into CHO, HeLa, and retinal pigment epithelial cells, primary human keratinocytes, and human conjunctival epithelial cells, occurs via pH-dependent or independent endocytosis [57–59]. Similarly, HCMV entry into different cell types may rely on different cellular receptors and routes, as illustrated for epithelial cells versus fibroblast cells [21,32,43]. In this study, APMAP was identified as a novel modulator in the early stage of HCMV infection. APMAP deficiency caused significantly lower HCMV infection in both epithelial and fibroblast cells (**Figs 2 and 3 and S3 and S4 Figs**) while over-expression of APMAP increased HCMV infection of less susceptible cells (**Figs 4 and 5 and S5**–**S7 Figs**), all in a pentamer non-specific manner. APMAP had a higher binding to gH/gL containing glycoprotein complexes at low pH than at neutral pH (**Fig 6A and 6B**). APMAP may also interact with viral tegument protein pp65 (**Fig 6D and 6F**). Although no significant decrease in HCMV attachment and viral internalization was observed between APMAP deficient versus wildtype cells (**Fig 7** and **S13 Fig**), APMAP deficiency was correlated with lower nuclear translocation of pp65 and lower IE mRNA level at the early stage of HCMV infection (**Figs 8 and 9**). Taken together, our results implicate APMAP as a broad modulator at early stage HCMV infection.

Phosphorylated tegument protein pp65 is a primary component of HCMV particle [8]. Nuclear translocation of viral tegument delivered pp65 occurs shortly after virus entry into the cytoplasm and does not depend on other viral proteins [60]. pp65 has been reported to modulate host immune responses to facilitate HCMV replication in multiple ways [61]. Yet the function of pp65 throughout viral life cycle is still poorly understood. Deletion of pp65 from AD169 does not significantly affect virus growth and morphogenesis in fibroblast cells, which suggests that pp65 is dispensable for virus replication in fibroblast cells [62]. However, specific modification of pp65 was shown to inhibit HCMV infection of fibroblast cells [63]. Also, pp65 is shown essential for viral replication in monocytes-derived macrophages [64]. Nuclear translocation of HCMV tegument protein pp71, also called pUL82, has been reported to stimulate viral IE protein expression and initiate the lytic viral infection cycle. Failed nucleus translocation of pp71 can lead to silence of IE protein expression and promote the latent viral infection cycle [65]. In our study, APMAP deficiency led to a substantial decrease in viral IE mRNA transcription at 6 h post viral infection and also significant reduction of late viral protein expression (**Figs 3 and 4**), consistent with the notion that APMAP may modulate HCMV infection at early stage, probably prior to transcription of IE gene. A potential interaction between APMAP and pp65 was demonstrated by biochemical assays (**Fig 7D–7F**). Efficient nuclear translocation of pp65 at early stage HCMV infection was detected in wildtype ARPE-19 and MRC-5 cells but not in APMAP deficient cells (**Figs 8 and 9**). This result suggests a role for APMAP in pp65 nuclear translocation after virus internalization. Whether pp65 nuclear translocation has similar regulatory role as pp71 in HCMV infection is an interesting topic to pursue.

The APMAP K/O in ARPE-19 cells in our study was not ideal; it caused only a two amino acid deletion (residues 77–78) near the transmembrane domain of APMAP. However, the deletion resulted in over 70% decrease of APMAP mRNA and protein expression (**Fig 2A–2C**). Furthermore, results of multiple experiments demonstrated significantly reduced HCMV infection in the ARPE-19 cells with defect APMAP expression. These experiments included monitoring viral infection indicated by GFP expression, viral protein detection at a late stage infection by western blot (**Fig 2D–2F**), and viral IE and pp65 mRNA level assessment at an early stage of infection (**Fig 4C and 4D**). These results indicate the importance of APMAP in HCMV infection of ARPE-19 cells. To further confirm the role of APMAP in HCMV infection, APMAP expression was silenced in two human epithelia-derived cell lines, HepG2 and Hela, and human fibroblast cells (MRC-5). Consistent with the results in APMAP K/O ARPE-19 cells, knockdown of APMAP expression significantly reduced HCMV infection in HepG2 and Hela cells (**S3 and S4 Figs**) and HCMV infection in MRC-5 cells (**Fig 3**). Thus, APMAP is necessary for HCMV infection in multiple cell lines. Further studies are needed to address the question of whether APMAP is important for HCMV infection of monocytes, myeloid lineage cells, or other primary cells.

HCMV is strictly a human pathogen. One hypothesis suggests that viral entry is one point at which there is a block against cross-species infection. Our data support this hypothesis; there was minimal viral entry in MDCK and NIH/3T3 cells. Interestingly, over-expression of APMAP not only enhanced HCMV infection in human cells (**Fig 4 and S6 Fig**) but also promoted HCMV entry in less susceptible canine MDCK and murine NIH/3T3 cells (**Fig 5 and S7 Fig**). Enhanced HCMV entry led to increased IE mRNA transcription in the APMAP O/E cells (**Fig 4C, Fig 5F and 5G, S6C Fig and S7F and S7G Fig**), which is a prerequisite for initiation of lytic infection cycle [65].

APMAP is a type II transmembrane protein with cellular localizations at both plasma membrane and endoplasmic reticulum (ER) in adipocytes [66,67]. APMAP plays a role in adipocyte differentiation [67–69]. It is associated with pathogenesis of gestational diabetes [70] and liver-specific metastasis in patients with colorectal cancer [71]. APMAP has also been reported as an endogenous suppressor of Aβ production in brain [72]. To our knowledge, no role has been described for APMAP in viral infection. The interaction of APMAP with HCMV may involve both gH/gL containing glycoproteins and pp65. Soluble pentamer and gH/gL dimer had stronger binding to APMAP at low pH (**Fig 6A and 6B**). This result is consistent with the identification of APMAP by pentamer pull-down at low pH (**S1B Fig**). The stronger binding of gH/gL to APMAP at low pH partially explains the influence of APMAP on laboratory-adapted strain with no pentamer expression (**Figs 2–5** and **S6 and S7 Figs**). The gH/gL has been reported to exist on HCMV virion as different functional glycoprotein complexes including gB-gH/gL, gH/gL/gO and gH/gL/pUL128-131 [73,74], which underlines the importance of gH/gL in HCMV infection. The fact that interaction between APMAP and gH/gL is enhanced at low pH makes it possible for APMAP to play a role in low-pH dependent HCMV membrane fusion. We observed a potential interaction between APMAP and pp65 (**Fig 6C–6F**). This prompted further examination, which revealed that nuclear translocation of pp65 was delayed in APMAP deficient cells (**Figs 8 and 9**). The impact of delayed pp65 nuclear translocation at early stage HCMV infection is unknown. APMAP may influence pp65 nuclear translocation directly. Alternatively, APMAP may influence pp65 nuclear translocation indirectly through low-pH dependent viral membrane fusion. Further investigations are needed to identify the mechanism by which APMAP affects low pH-dependent or -independent HCMV infection.

In summary, APMAP was identified as a novel modulator active at an early stage of HCMV infection. APMAP could have a role in HCMV infection through interaction with gH/gL containing glycoprotein complexes at low pH or through mediating nucleus translocation of pp65. Further efforts are needed to clearly define the mechanism of APMAP in HCMV infection of different cells.

## Materials and methods

### Cells, virus strains and reagents

Human retinal pigment epithelia cells ARPE-19 (ATCC, CRL-230) were cultured in DMEM/F12 (50/50) plus 10% FBS. Human MRC-5 (ATCC, CCL-171) embryonic lung fibroblast cells, dog kidney epithelial cells MDCK (ATCC, CCL-34) and mouse embryo fibroblast NIH/3T3 cells (ATCC, CRL-1658) were cultured in DMEM plus 10% FBS. The generation of HCMV strains, AD169-GFP, AD169rev, and AD169rev-GFP were described previously [48,50]. Growth curves of AD169-GFP and AD169rev-GFP were determined by infection of both ARPE-19 cells and MRC5 cells at MOI = 0.1. The infected culture supernatants were collected at indicated times post infection, and the viral titers were determined by standard TCID_50_ assay. To construct HSV-2 with GFP reporter, a CMV promoter derived GFP-expressing cassette was inserted between UL37 and UL38 of the genome of HSV-2 (MS strain) using CRISPR/Cas9 technology. The sgRNA was designed to avoid disrupting the reading frames of any ORFs. The reporter virus with GFP expression was grown in ARPE-19 cells and subjected to several rounds of plaque purification before use. Growth curve analysis showed that insertion of GFP-expressing cassette did not compromise the growth of HSV-2 in ARPE-19 cells as compared with parental virus (Data not shown). The HSV-2 virus with GFP reporter was titrated by standard TCID50 assay.

HCMV pentamer specific human antibody 2–25 and gH specific rabbit antibody (223.4) were described previously [46]. HCMV gO specific rabbit polyclonal antibodies were generated in house. Briefly, gO (pUL74 of AD169, amino acid 31–466) with a C-terminal 6X his tag was expressed in *E*. *coli*. Recombinant gO was solubilized from inclusion bodies and purified to 85% purity. New Zealand White rabbits were immunized three times and the immune sera were screened for anti-gO activity in ELISA. Anti-gO antibodies were purified by protein G from the pooled rabbit serum. Rabbit anti-gL antibodies and rabbit anti-APMAP antibodies were generated in a same way. IE specific mouse mAb (clone L-14) was obtained from ATCC (HB6554). Odyssey blocking buffer (Cat#: 927–40000), IRDye 800CW Streptavidin (Cat#: 926–32230) and Sapphire700 (Cat#: 928–40022) were from LI-COR Biosciences. Li-DraQ5 (Cat#: 4084S) was from Cell Signaling. A mouse mAb 4F6 that recognizes APMAP was obtained from OriGene (Cat#: TA504220). His-tagged pp65 protein (Cat#: Ab43041), a mouse mAb that recognizes pp65 (Cat#: Ab6503), and rabbit polyclonal antibodies that recognize EEA1 (Cat#: ab2900) were obtained from Abcam. Flag tag specific mouse mAb (Cat#: F3165) and β-actin specific mouse mAb (Cat#: A5316) were obtained from Sigma. HRP conjugated mouse anti-His tag mAb was obtained from R&D (Cat#: mab050h).

### SDS-PAGE and Western blot assay

Protein samples were separated on pre-cast 10% SDS-PAGE gels purchased from BioRad. The proteins on the gel were either stained with coomassie blue R-250 or transferred to nitrocellulose membrane for blotting. The membrane was blocked in 5% non-fat milk in PBST (PBS containing 0.05% Tween-20) at room temperature (RT) for 2 h followed by incubation with indicated primary antibodies at RT for 2 h. After washing with PBST 3 times (10 min/each), the membrane was incubated with corresponding horseradish peroxidase (HRP)-conjugated secondary antibodies at RT for 1 h. After washing 5 times (5 min/each) with PBST, chemiluminescent HRP subtracts solution was added to the membrane for development. The digital image was captured on a FluorChem M machine.

### Protein expression and purification

Soluble gH/gL dimer and pentamer (gH/gL/pUL128-131) glycoprotein complexes were expressed in CHO cells, purified and characterized to have > 95% purity as described previously [45]. The gH subunit of gH/gL and pentamer were designed with a truncation of its cytoplasmic and transmembrane domains. They have a strep and His-tag fused at the C-terminus via a flexible linker to facilitate purification and detection. For generation of APMAP-Fc, the coding sequence of human APMAP was amplified from cDNA of ARPE-19 cells. The gene for the extracellular domain of APMAP (amino acids 62–416) was amplified and inserted into a vector containing expression cassette with a C-terminal Fc tag of mouse IgG2a, which binds both protein A and protein G. The construct was confirmed by sequencing before transfection into Expi293 cells for protein expression. APMAP-Fc was purified from the culture medium of transfected cells using protein-A resin. The purified protein was confirmed by SDS-PAGE/coomassie blue staining assay and quantified by Nanodrop-2000.

### Pulldown of membrane proteins by pentamer

Membrane proteins were extracted from ARPE-19 cells using a BioVision (Mountain View, CA) plasma membrane protein extraction kit according to the manufacturer’s instructions. The concentration of extracted membrane proteins was determined using BCA Protein Assay Kit according to the manufacturer’s instructions (Thermo Fisher Scientific). For pull-down assay, soluble pentamer was mixed with extracted membrane proteins in citric acid buffer (pH 5.5) at a final concentration of 30 μg/ml and incubated at room temperature for 1 h. Then, the pH of the mixtures was adjusted to neutral using 1 M Tris-HCl buffer. The pentamer in the mixture was pulled down by protein A/G Magnetic Beads (Thermo Fisher Scientific) which were pre-incubated with pentamer specific antibody 2–25. After extensive washing, the pull-down proteins were separated on SDS-PAGE gel and analyzed by mass spectrometry analysis.

### Generation of stable cell lines

The 23 target genes identified from the pull-down assay were knocked out individually in ARPE-19 cells using a CRISPR/Cas9 system. ARPE-19 cells stably overexpressing Cas9 (ARPE-19-Cas9) were generated by Lentivirus (Purchased from Sigma) transduction and selection under 40 μg/ml of blasticidin for 2 weeks. Overexpression of Cas9 in ARPE-19 cells was confirmed by western blot assay using Cas9 specific antibody (Data not shown). Then, the ARPE-19-Cas9 cells were infected with lentivirus particles containing the 23 targets specific sgRNAs (designed and made by Sigma) separately and cultured for selection with 2 μg/ml of puromycin plus 40 μg/ml blasticidin for two weeks. The resulting puromycin and blasticidin resistant mixture cells were not confirmed further before use in screening of HCMV infection. ARPE-19 cells expressing Cas9 and a control sgRNA that does not target any genes were used as vector control. The target sequence of all sgRNA are shown in **S1 Table**.

Overexpression of APMAP in ARPE-19, MDCK, and NIH/3T3 cells was achieved by infecting the cells with lentiviral particles carrying full length human APMAP ORF with a c-terminal Myc/Flag tag (purchased from OriGene) and then cultured for selection with puromycin for at least two weeks. Overexpression of APMAP in the cells was confirmed by western blot assay using flag tag specific antibody before use. Cells infected with lentivirus particles containing empty vector were used as vector controls.

For knockdown of APMAP, five validated APMAP targeting shRNA constructs based on pLKO.1-puro vector were purchased from Sigma. Lentivirus particles were prepared in house through co-transfection of 293T cells with pLKO.1-puro vector, pCMV-Δ8.9 and pVSV-G plasmids. Knockdown of APMAP in MRC-5, HepG2 and Hela cells was achieved by infecting the cells with lentivirus particles carrying APMAP specific shRNA and then selected under puromycin at appropriate concentrations for at least two weeks. Cells expressing a scramble shRNA were used as vector controls. The shRNA target sequences are shown in **S1 Table**.

### In-Cell western assay

A high throughput neutralization assay was used with minor modification to determine HCMV entry in ARPE-19 cells through IE staining [49]. Briefly, ARPE-19 and the 23 knockout cells were seeded in 96-well plate (1.4×10^4^ cells/well) and cultured overnight to reach ~ 90% confluence. The cells were infected with AD169rev at a MOI = 0.1 with at triplicate wells. About 22 h after infection, the medium was gently removed and the cells were fixed by addition of 100 μl/well of 3.7% formaldehyde at room temperature for 20 min without shaking. After removal of the fixing solution, the cells were washed with 200 μl/well of 0.1% Triton X-100/PBS for five times with 4 min interval incubation at RT between washes. The plate was blocked using 100 μl/well of Odyssey blocking buffer for 1h at RT with gently rotation. After each of the following steps, the plate was washed 5 times with PBS plus 0.05% Tween-20 and all the antibodies were diluted in Odyssey Blocking Buffer plus 0.05% Tween-20. The plate was reacted with 50 μl/well of HCMV IE specific L-14 mAb (2 μg/ml) at RT for 2 h followed by 50 μl/well of biotinylated anti-mouse IgG at RT for 1 h. Then, 50 μl/well of a cocktail containing IRDye 800CW Streptavidin (1:1000 final dilution), Sapphire700 (1:1000 final dilution), and 5 mM DraQ5 solution (1:10,000 final dilution) was added to the plate and incubated at RT for 1 h. After the final wash, the plate was air-dried in the dark for 10–20 min and scanned using a Li-Cor Aerius IRDye plate reader. The fluorescence intensities at 800 nm and 700 nm were collected and the 800/700 ratio were used to calculate the relative % of IE-1 expression.

### HCMV infection, imaging and quantification of GFP positive cells

The cells were seeded in a 96-well plate and then infected after a day with AD169rev-GFP or AD169-GFP at indicated MOI for three or four replicate wells. 4 h after infection, virus containing medium was replaced with fresh medium and the cells were cultured at 37°C incubator for 48 or 72 h. The cells in each well were imaged under a LEIKA fluorescence microscopy to see GFP expression. For quantitation of GFP positive cells, single-well images of the 96-well plate were captured by C.T.L. Immunospot machine using Immunospot 7.0 Pro FluoRo-X suite software under fluorescence cell mode for GFP. The number of GFP positive cells in each well were counted automatically using the “Count” module of the software under a basic count mode.

### Flow cytometry assay

ARPE-19 cells, MRC-5 or MDCK cells were detached from culture dish using an enzyme free cell disassociation solution. After blocking with 3% BSA in PBS for 30 min, pentamer was incubated with cells at a final concentration of 20 μg/ml in either neutral pH (7.4) or low pH (5.5) citric buffer for 1 h on ice. After washing away of unbound pentamer with PBS, the cells were stained with FITC-conjugated anti-His tag antibodies for 1 h on ice. The cells were extensively washed before detection on a Guava easycyte HT machine. Cells with no pentamer incubation but stained with the FITC-conjugated anti-His tag antibodies were used as negative control for gating.

### Immunofluorescence assay

ARPE-19 cells grown in chamber slides were fixed by 4% PFA for 20 mins at RT and then blocked with 1% BSA for 1h. For detection of pentamer binding on ARPE-19 cells, soluble pentamer was incubated with ARPE-19 cells at indicated concentrations in 0.1% BSA in PBS for 1 h at 37°C. The cells were washed 3 times with PBS to remove unbound pentamer and then stained with FITC-conjugated anti-His tag antibody which recognizes gH subunit of the pentamer. Nucleus was stained with DraQ5. Cells incubated with buffer only were stained in the same way and used as negative control. The cells were imaged using a LEIKA confocal microscopy.

For detection of internalization of HCMV, the MRC-5 cells (wildtype and APMAP K/D cells) were seeded in chamber slides (1×10^4^ cells/well) a day in advance. The cells were pre-cooled at 4°C before adding 2.4×10^6^ PFU/well of AD169rev for attachment at 4°C for 1 h to synchronize virus infection. Then, the cells were transferred to 37°C and cultured for 0.5 h or 4 h before fixation with 4% PFA and permeabilized with 0.05% NP-40 in PBS. The cells were blocked with 1% BSA and double stained with mouse anti-pp65 and Rabbit anti-EEA1 antibodies for 1 h at room temperature. After washing with PBS for 3 times, 1/1000 diluted cross adsorbed AlexaFluo 488 conjugated goat anti-Mouse IgG antibodies and Texas red conjugated goat anti-rabbit IgG antibodies were added to the cells and reacted at room temperature for 1 h. Nuclei were stained by 1/1000 diluted DraQ5 for 30 min at room temperature. The images were taken under a LEIKA confocal microscopy and processed using LAS AF lite software at same parameters. HCMV internalization into wildtype and APMPA K/O ARPE-19 cells were detected as described above except that only pp65 specific antibodies were used to stain the cells and the images were captured using an Olympus fluorescence microscope.

### Pull down of HCMV virion by APMAP-Fc

1 μg APMAP-Fc was incubated with 2.58×10^6^ PFU of AD169rev diluted in 500 μl PBS. Control CD47-Fc protein was incubated with 2.58×10^6^ PFU of AD169rev diluted in RIPA buffer (50 mM Tris-HCl, 150 mM NaCl, 1% NP-40, 0.1% SDS, 0.5% sodium deoxycholate, 5 mM EDTA). These incubations were carried out for 2 h at 4°C with gentle rotation. Dynabeads Protein G was added to the mixtures and incubated with gentle rotation at 4°C for 1 h. The beads were washed 3 times with PBST and suspended into SDS containing loading buffer before boiling at 100°C for 5 min. The samples were separated on 10% SDS-PAGE and analyzed by western blot assay using rabbit anti-gH (Mab 223.4) and mouse anti-pp65 antibodies. APMAP-Fc and CD47-Fc were detected by HRP-conjugated goat anti-mouse IgG antibodies.

### ELISA assay

APMAP-Fc and a control CD47-Fc in PBS (4 μg/ml) were coated (50 μl/well) in Costar 96-well high binding plate overnight at 4°C. The coating antigens were removed. The plate was blocked with 200 μl/well of 5% non-fat milk in PBST for 1 h at 37°C. 50 μl/well of soluble pentamer or gH/gL dimer diluted in PBS (pH7.0) was added to the plate at indicated concentrations for reaction with APMAP-Fc. 50 μl/well of soluble pentamer or gH/gL dimer diluted in citric acid buffer (pH5.5) at indicated concentrations was added to the plate for reaction with CD47-Fc. Plates were incubated at 37°C for 1h. After washing with PBST 5 times, bound pentamer or gH/gL dimer were detected with HRP conjugated anti-His tag antibodies which recognizes gH subunit at 37°C for 1h. After washing with PBST 5 times, TMB substrate mixture was used for color development. Absorbance at 450nm was read on a Molecular Devices Spectra Max M4 machine.

### RT-qPCR assay

Total RNA was extracted from cells with or without HCMV infection using Trizole (Invitrogen). Then, 1 μg RNA was reverse transcribed to cDNA using iScript TM Select cDNA synthesis kit (Biorad, Catalog No.: 170–8896). The resulting cDNA was used as template for qPCR using BioRad SsoAdvanced^TM^ Universal SYBR^®^ green supermix kit and BioRad CFX96 C1000 thermal cycler system. The primers for APMAP are APMAP-RT-F: CATTGCCCGGTTTGGTTCG and APMAP-RT-R: CACTTCACGTTTCCAGGGATTTA; for HCMV IE are IE-RT-F: TCTGCCAGGACATCTTTCTCG and IE-RT-R: GGAGACCCGCTGTTTCCAG; for pp65 are pp65-RT-F: GCAGAACCAGTGGAAAGAGC and pp65-RT-R: CAGCGTGACGTGCATAAAGA; for internal control GAPDH are GAPDH-RT-F: GAAGGTGAAGGTCGGAGTC and GAPDH-RT-R: GAAGATGGTGATGGGATTTC. The cycling conditions consisted of a denaturation step at 95°C for 30 s and 40 cycles of 95°C for 5 s and 60°C for 30 s. Data analysis was performed using the 2^-ΔΔCT^ method. The CT values of all samples were first normalized with GADPH and then compared to that of the wildtype cells.

### HCMV attachment assay

The cells were seeded into a 24-well plate (1×10^5^ cells/well) one day in advance and pre-cooled at 4°C for 10 mins. After removing the medium, 2.4×10^6^ PFU/well of AD169rev diluted in 200 μl medium was added to the cells and incubated at 4°C for 90 min. The unbound virus was removed by washing with cold PBS for 3 times. Then, the cells were lysed in SDS containing buffer for western blot assay using indicated antibodies, or subjected to total DNA extraction and qPCR for quantification of HCMV genome. Specifically, cell genomic DNA and HCMV genome were extracted together using a QIAamp DNA blood mini kit. The DNA samples were quantified by Nanodrop-2000 and adjusted to same concentration. The same amount of DNA was used as template for qPCR using BioRad SsoAdvanced^TM^ Universal SYBR^®^ green supermix kit and BioRad CFX96 C1000 thermal cycler system as described above. The primers used for HCMV genome detection are as described above. A plasmid with pp65 gene insert, pET28a-pp65, was used to generate standard curve for calculation of HCMV genome copies in unknown samples. Cells incubated with medium only were used as negative control.

### Bio-layer interferometry assay

The binding of pentamer and gH/gL dimer to APMAP-Fc was detected by bio-layer interferometry (BLI) assay on an Octet RED 96 system (ForteBio). After a brief rinse in kinetics buffer, the protein A biosensor tips were dipped into 20 μg/ml of APMAP-Fc or CD47-Fc in kinetics buffer for 10 min. Following a 60 sec rinse in kinetics buffer, the APMAP-Fc or CD47-Fc loaded sensors were allowed to associate with pentamer or gH/gL dimer or pp65 at indicated concentrations for 10 min and then dissociate in kinetics buffer for 10 min. The APMAP-Fc or CD47-Fc loaded sensors were also allowed to associate with kinetics buffer alone to serve as reference and subtracted before data analysis.

### PEG treatment assay

MRC-5, sc-shRNA control and the APMAP K/D cells were seeded in 96-well cell culture plate a day in advance. AD169rev-GFP and AD169-GFP were added to the cells at a MOI = 1.0 and cultured at 37°C for 1 h to allow virus infection. The uninfected virus was removed by washing with warm PBS twice. Then, the cells were treated with 150 μl/well of pre-warmed 44% (W/V) PEG-8K for 30 sec and followed by washing with 200 μl/well of warm PBS 4 times to remove PEG-8K. After that, fresh medium was added to cells and the plate was incubated for 48 h. Then, a C.T.L. Immunospot analyzer using Immunospot 7.0 Pro FluoRo-X suite software was used to capture images under fluorescence cell mode for GFP. The number of GFP positive cells in each well was counted automatically using the software under a basic count mode.

### Statistical analysis

Data were plotted and analyzed using GraphPad Prism® 8. The unpaired two-tailed student t-test was used to determine the significance of differences between pairs of samples as indicated in figures or figure legend of Figs 1–5, 7 and 9, supplementary Figs 2–8 and 11–13. Two-way ANOVA analyses was used to compare the growth rates of vector control, APMAP K/O (K/D), or APMAP O/E cells with the corresponding wildtype cells in supplementary Fig 9. Statistical significance was indicated as follows: n.s., P> 0.05; *, P<0.05; **, P<0.01; ***, P<0.001; or ****, P<0.0001. All results are presented as mean values ± standard deviation (SD).

## Supporting information

S1 Fig (**A-B**) Binding of soluble pentamer to (**A**) MRC-5 cells and (**B**) MDCK cells. 2×10^5^ cells per sample of suspended MRC-5 or MDCK cells were blocked with 3% BSA in PBS for 30 min and then incubated with 200 μl soluble pentamer diluted in PBS at a concentration of 25 μg/ml for 1 h on ice. The cells were washed to remove unbound pentamer and followed by staining with FITC-conjugated anti-His tag antibodies for 1 h on ice. The cells were washed extensively before detection on a Guava easycyte HT machine. Cells incubated in buffer without pentamer but stained with the FITC-conjugated anti-His tag antibodies served as negative control. (**C**) A diagram showing the procedure for pull-down assay. ARPE-19 cells membrane proteins were extracted and mixed with recombinant pentamer protein at 30 μg/ml in citric acid buffer (pH 5.5) and incubated at room temperature for 1 h. Then, the mixtures were adjusted to neutral pH using 1M Tris-HCl buffer. The pentamer in the mixture was pulled down by a pentamer-specific antibody 2–25 along with Protein A/G Magnetic Beads. The pull-down proteins were separated on SDS-PAGE and analyzed by mass spectrometry. (**D**) 23 membrane proteins were chosen from the list of proteins identified by mass spectrometry assay using membrane protein with extracellular domain as criteria.
(TIF)

S2 Fig Viral growth curves and the effects of MOI on HCMV infection of APMAP K/O cells. (**A**) Single step growth curves of AD169-GFP and AD169rev-GFP in MRC-5 or ARPE-19 cells. The infectious viral particles were measured in TCID50 assays. (**B**) Wildtype ARPE-19, vector control and APMAP K/O cells cultured in 96-well plate were infected with AD169rev-GFP at indicated MOIs. Four replicate wells were infected at each MOI. 72 h later, the plate was read by C.T.L. Immunospot machine to capture images under fluorescence cell mode for GFP. GFP positive cells in each well were counted automatically. The data are shown as relative percentages of the number of GFP positive cells to that of infected wildtype ARPE-19 cells at same MOI. The relative % of GFP+ cells in vector control and APMAP K/O cells were compared individually to that of wildtype ARPE-19 cells at same MOI using unpaired two-tailed student t-test for significance analysis.
(TIF)

S3 Fig APMAP knockdown reduced AD169rev-GFP entry into HepG2 cells. (**A**) APMAP knockdown in HepG2 cells was achieved by infecting HepG2 cells with lentivirus expressing APMAP-specific shRNA under puromycin selection. APMAP protein expression in the stable knockdown cells were detected by western blot assay using APMAP specific mAb 4F6, β-actin served as loading control. (**B-D**) Wildtype HepG2 and the APMAP knockdown cells were infected with AD169rev-GFP at indicated MOIs in 96-well plate. (**B**) The plate was read by C.T.L. Immunospot to capture images under fluorescence cell mode for GFP at 48 h after infection. GFP positive cells in each well were counted automatically using the software. The data are shown as relative percentages of the number of GFP positive cells to that of infected wildtype HepG2 cells. The bars represent means ± SD for four replicate wells. (**C**) Representative images showing overall GFP positive cells in infected (MOI = 2.0) wildtype and APMAP knockdown HepG2 cells. Images were captured using an Olympus fluorescence microscope. Bar = 100 μm. (**D**) The cells were collected at 2 days after infection for qRT-PCR detection of viral IE mRNA. GAPDH mRNA served as internal control. Data analysis was performed using the 2^-ΔΔCT^ method. The data are shown as relative percentages of IE mRNA level to that of infected wildtype HepG2 cells. The black bars represent means ± SD for triplicate wells. The relative % of GFP positive cells or relative IE mRNA (%) in sc-shRNA or shAPMAP treated cells were compared individually to that of wildtype HepG2 cells infected at same MOIs using unpaired two-tailed student t-test for significance analysis.
(TIF)

S4 Fig APMAP knockdown reduced AD169rev-GFP entry into HeLa cells. (**A**) APMAP knockdown in HeLa cells was achieved by infection with lentivirus particles expressing APMAP-specific shRNA under puromycin selection. APMAP protein expression in the stable knockdown cells was detected by western blot assay using APMAP specific mAb 4F6, β-actin served as loading control. (**B-D**) Wildtype HeLa and the APMAP knockdown cells were infected with AD169rev-GFP (MOI = 1.0) in 96-well plate. (**B**) The plate was read by C.T.L. Immunospot machine at 48 h after infection and GFP positive cells in each well were counted automatically using the software. The data were shown as the number of GFP positive cells per well. The black bars represent means ± SD for four replicate wells. (**C**) Representative images showing overall GFP positive cells in wildtype and APMAP knockdown HeLa cells. Images were captured using Olympus fluorescence microscopy. Bar = 100 μm. (**D**) The cells were collected at 2 days after infection for qRT-PCR detection of viral IE mRNA. GAPDH mRNA served as internal control. Data analysis was performed using the 2^-ΔΔCT^ method. The data are shown as relative percentages of IE mRNA level to that of infected wildtype Hela cells. The black bars represent means ± SD for triplicate wells. The number of GFP positive cells, and relative IE mRNA (%) in sc-shRNA and shAPMAP expressing cells were compared individually to that of wildtype HeLa cells using unpaired two-tailed student t-test for significance analysis.
(TIF)

S5 Fig APMAP knockdown reduced HCMV infection of MRC-5 cells. Wildtype and APMAP K/D MRC-5 cells were infected with (**A-B**) AD169rev-GFP and (**C-D**) AD169-GFP at a MOI = 1.0 in 96-well plate, respectively. The cells were collected for RNA extraction and RT-qPCR to detect viral (**A, C**) IE and (**B, D**) pp65 mRNA level at 72 after infection. GAPDH mRNA served as internal control. Data analysis was performed using the 2^-ΔΔCT^ method. The data are shown as percentages of IE or pp65 mRNA level in infected knockdown cells relative to that of infected wildtype MRC-5 cells. The black bars represent means ± SD of four wells. The relative IE mRNA (%) in sc-shRNA or shAPMAP expressing cells were compared individually to that of wildtype MRC-5 cells using unpaired two-tailed student t-test for significance analysis.
(TIF)

S6 Fig Enhanced AD169-GFP infection in APMAP O/E ARPE-19 cells. (**A**) APMAP K/O, O/E and wildtype ARPE-19 cells grow in 96-well plate were infected with AD169-GFP (MOI = 1.0), respectively. The cells were imaged under Fluorescence microscopy to see GFP expression at 2 days post infection using Olympus Fluorescence microscopy. Bar = 100 μm. (**B**) The plate was read by C.T.L. Immunospot machine at 48 h after infection and GFP positive cells in each well were counted automatically using the software. The data were shown as the number of GFP positive cells per well. The black bars represent means ± SD for four wells. (**C**) The cells were collected at 2 days after infection for qRT-PCR detection of viral IE and pp65 mRNA. GAPDH mRNA served as internal control. Data analysis was performed using the 2^-ΔΔCT^ method. The data are shown as relative IE or pp65 mRNA level to that of infected wildtype cells. The black bars represent means ± SD for triplicate wells. The number of GFP positive cells, relative IE mRNA and relative pp65 mRNA in vector control, APMAP K/O and APMAP O/E cells were compared individually to that of wildtype ARPE-19 cells using unpaired two-tailed student t-test for significance analysis in S6B and S6C Fig. (**D**) Determination of HCMV protein expression of AD169-GFP infected cells at 6 days post infection by western blot assay using anti-pp65 and anti-gH antibodies. β-actin served as loading control.
(TIF)

S7 Fig Overexpression of APMAP enhanced HCMV entry in NIH/3T3 cells. (**A**) APMAP O/E stable NIH/3T3 cells were established with lentivirus particles carrying APMAP expression cassette with full length sequence with a Myc/Flag tag at its C-terminus. APMAP overexpression in NIH/3T3 cells were confirmed by western blot analysis using mouse anti-Flag tag or anti-APMAP (4F6) antibodies. β-actin served as loading control. (**B-F**) NIH/3T3 wildtype and APMAP O/E stable cells were infected with AD169rev-GFP and AD169-GFP at MOI = 1.0 in 96-well plate, respectively. (**B-C**) The plate was read by C.T.L. Immunospot machine to capture images under fluorescence cell mode for GFP at 2 days post infection. GFP positive cells in each well were enumerated automatically using the software. The data were shown as means ± SD of the number of GFP positive cells of four replicate wells. (**D**-**E**) Representative images (Bar = 100 μm) showing overall GFP positive cells at day 3 post infection by (**D**) AD169rev-GFP or (**E**) AD169-GFP were captured by Olympus fluorescence microscopy. (**F-G**) The cells were collected at 2 days after infection for qRT-PCR detection of viral IE and pp65 mRNA. GAPDH mRNA served as internal control. Data analysis was performed using the 2-ΔΔCT method. The data are shown as relative IE or pp65 mRNA level to that of infected wildtype cells. The black bars represent means ± SD for triplicate wells. The number of GFP positive cells, relative IE mRNA and relative pp65 mRNA in vector control and APMAP O/E cells were compared individually to that of wildtype NIH/3T3 cells using unpaired two-tailed student t-test for significance analysis in S7B, S7C, S7F and S7G Fig.
(TIF)

S8 Fig HCMV infection in MDCK and NIH/3T3 cells. Wildtype, vector control, and APMAP O/E MDCK or NIH/3T3 cells were seeded (1.5×10^5^ cells/well) in a 24-well plate. The next day, the cells were infected with AD169rev-GFP at a MOI = 1.0 for 3 h at 37°C. Virus containing medium was removed and 1 ml of fresh medium was added to each well. After 4 days of culture, the cells were harvested. (**A-B**) Western blot analysis was performed for detection of viral protein pp65. β-actin served as loading control. The cell culture medium was centrifuged at 10,000×g for 5 mins to remove cell debris. 100 μl supernatant was added to one well of MRC-5 cells grown in a 96-well plate for detection of virus release. 48 h later, images of GFP positive cells among infected MRC-5 cells were captured using a C.T.L. Immunospot machine and counted using the instrument’s software. (**C-D**) Titer of infectious virion released from infected MDCK and NIH/3T3 cells. One GFP positive cell was counted as one PFU. Black bars represent means ± SD for three replicate wells. The titer of infectious virion produced by vector control and APMAP O/E cells were compared individually to corresponding wiltype cells using unpaired two-tailed student t-test for significance analysis. (**E-F**) Representative whole-well images of culture medium infected MRC-5 cells. Infection experiments were performed in triplicate wells. Data were representative results of two independent experiments.
(TIF)

S9 Fig Growth curve of the APMAP modified cells. The APMAP K/O, K/D or O/E (**A**) ARPE-19 cells, (**B**) MRC-5 cells, (**C**) HepG2 cells, (**D**) Hela cells, (**E**) NIH/3T3 cells and (**F**) MDCK cells were seeded in 96-well culture plate at 2000 cells per well and cultured for 3–5 days. AlamarBlue solution was added to the cells shortly after seeding and then every 24 h. Fluorescence intensity was measured at 4 h post addition of alamarBlue as instructed by the manufacture. At each time point, 6 replicate wells were detected for each cell line. Data were shown as mean ± SD of fluorescence intensity. Two-way ANOVA analyses was used to compare the growth rates of vector control, APMAP K/O (K/D), or APMAP O/E cells with the corresponding wildtype cells.
(TIF)

S10 Fig Uptake of AlexaFluo-488-transferrin by cells. (**A**) ARPE-19 wt, APMAP K/O and APMAP O/E cells grown in chamber slides were pre-treated with 30 μm chlorpromazine (CPZ) or equal volume of solvent (DMSO) in serum free medium for 1h. Old medium was removed. 100 μl/well of AF488-Tfr (0.1 mg/ml) in fresh medium was incubated with the cells at 37°C for 30 min. The cells were washed once with citrate buffer (40 mM citric acid, 10 mM KCl, 135 mM NaCl, pH 3.0) and 3 times with PBS to remove cell surface AF488-Tfr before fixation with 4% paraformaldehyde and nucleus staining with DraQ5. Pictures were taken using a LEIKA confocal microscopy. Bar = 20 μm. (**B-G**) Quantification of AF488-Tfr uptake in APMAP K/O K/D or O/E (**B**) ARPE-19 cells, (**C**) MRC-5 cells, (**D**) HepG2 cells, (**E**) Hela cells, (**F**) MDCK cells and (**G**) NIH/3T3 cells by flow cytometry assay. Cells grown in 12-well plate were pre-treated with CPZ and incubated with AF488-Tfr as described in (**A**). After washing with citrate buffer to remove cell surface AF488-Tfr, the cells were suspended by treatment with trypsin and detected using on a Guava easycyte HT machine. Data were shown as mean fluorescent intensities of green signal for each samples.
(TIF)

S11 Fig The effect of APMAP on HSV-2 infection. The cells (1.4×10^4^ cells per well) were seeded in 96-well plate one day ahead infection. Cells with about 95% confluency were infected with HSV-2 GFP reporter virus at about 60 PFU/well for ARPE-19 cells, 300 PFU/well for MRC-5, HepG2 and Hela cells, 1500 PFU/well for NIH/3T3 and MDCK cells with at least three replicate wells. Medium was removed at 48 h post infection. The plate were imaged at same exposure time and gain using Immunospot 7.0 Pro FluoRo-X suite for GFP detection on a C.T.L. Immunospot machine. (**A**) Representative single-well images of infected cells. (**B-G**) GFP positive viral plaques in the wells were counted manually. Data were shown as mean ± SD of the number of GFP positive viral plaques. The number of GFP positive cells in vector control, APMAP K/O, K/D or O/E cells were all compared individually to that of corresponding wildtype cells using unpaired two-tailed student t-test for significance analysis.
(TIF)

S12 Fig (**A**) 10 μg of purified APMAP-Fc and a control CD47-Fc protein analyzed by SDS-PAGE and coomassie blue staining assay. (**B-C**) The binding of (**B**) pentamer and (**C**) gH/gL dimer to APMAP-Fc at indicated concentrations were detected by biolayer interferometry (BLI) assay using protein A sensors. The binding of kinetic buffer to APMAP-Fc loaded sensor were used as reference and subtracted before data analysis. (**D-E**) Inhibition of soluble pentamer binding to APMAP by gL or APMAP specific rabbit polyclonal antibodies. Costar 96-well high binding plates were incubated with APMAP-Fc (4 μg/ml in PBS, 50 μl/well) overnight at 4°C. Unbound APMAP-Fc was removed. The plate was blocked with 5% non-fat milk for 1 h. Soluble pentamer (final concentration 5 μg/ml) was incubated with purified rabbit anti-gL polyclonal IgGs, rabbit anti-APMAP polyclonal IgGs or rabbit anti-HSV polyclonal IgGs (as control IgG) at indicated concentrations in low pH buffer (150 mM Citric acid, 50 mM NaCl, pH 5.5) at room temperature for 30 mins. Pentamer incubated without antibody served as positive control. 50 μl/well of the mixture was added to APMAP coated plate and incubated at 37°C for 2 h. BSA diluted in low pH buffer served as negative control. Wells were tested in triplicate for each concentration. The plate was washed with PBST five times. HRP conjugated anti-His tag antibody (1:2000 dilution) that recognizes the gH subunit were added to plate for detection of soluble pentamer. The plate was washed with PBST five times then developed using TMB substrate mixture. Absorbance at 450nm was recorded on a Molecular Devices Spectra Max M4. Data are presented as mean values ± standard deviation (SD). The OD450nm values of all samples were compared individually to that of pentamer-only control using the unpaired two-tailed student t-test for significance analysis.
(TIF)

S13 Fig Inhibiting effect of APMAP-Fc on HCMV infection and PEG treatment assay in APMAP K/D MRC-5 cells. **(A)** 50 μl APMAP-Fc or CD47-Fc control were mixed with equal volume of AD169rev-GFP (about 100 PFU/well) at indicated concentrations and incubated at 37°C for 30 min before adding to pre-seeded ARPE-19 cells in 96-well plate. 2 hours later the virus mixtures were replaced with fresh medium. The cells were continue cultured for 3 days before quantitation of GFP positive cells in each well. Pairs of samples were compared inividually using the unpaired two-tailed student t-test for significance analysis. **(B-C)** Wildtype, sc-shRNA control and the APMAP K/D MRC-5 cells were seeded in 96-well plate 1 day before. (**B**) AD169rev-GFP and (**C**) AD169-GFP were added to the cells at a MOI = 1.0 and cultured at 37°C for 1 h. The uninfected virus was removed by washing with warm PBS for 2 times. Then, the cells were treated with150 μl/well of pre-warmed 44% (W/V) PEG-8K for 1 min and followed by washing with 200 μl/well of warm PBS for 4 times to remove PEG-8K. After that, fresh medium was added to cells and the plate was continue cultured for 48 h before read by C.T.L. Immunospot machine to capture images under fluorescence cell mode for GFP. The number of GFP positive cells in each well was counted using the software. The data are shown as means ± SD of the number of GFP positive cells in four replicate wells. The number of GFP positive cells in PEG8K treated cell lines were compared to corresponding non-treated cell lines using the unpaired two-tailed student t-test for significance analysis.
(TIF)

S1 Table Target host sequences for sgRNA and shRNA. (DOCX)
