# Supplementary figures and images for: Identification of adipocyte plasma membrane-associated protein as a novel modulator of human cytomegalovirus infection
Source: PLoS Pathog. 2019 Jul 29;15(7):e1007914. doi: 10.1371/journal.ppat.1007914 (PMC6687193; doi:10.1371/journal.ppat.1007914)

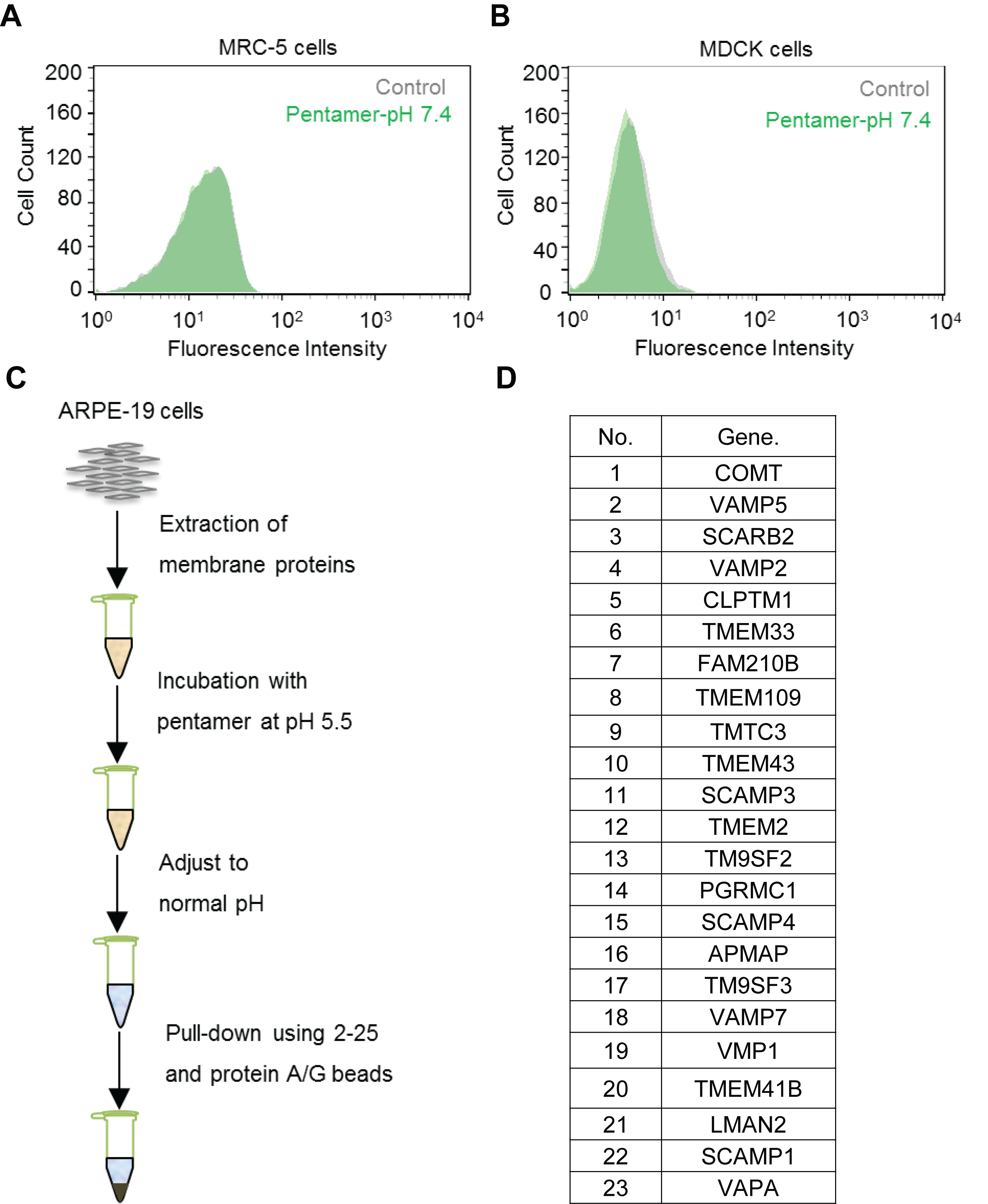

Supplement: S1 Fig — (A-B) Binding of soluble pentamer to (A) MRC-5 cells and (B) MDCK cells. 2×10^5^ cells per sample of suspended MRC-5 or MDCK cells were blocked with 3% BSA in PBS for 30 min and then incubated with 200 μl soluble pentamer diluted in PBS at a concentration of 25 μg/ml for 1 h on ice. The cells were washed to remove unbound pentamer and followed by staining with FITC-conjugated anti-His tag antibodies for 1 h on ice. The cells were washed extensively before detection on a Guava easycyte HT machine. Cells incubated in buffer without pentamer but stained with the FITC-conjugated anti-His tag antibodies served as negative control. (C) A diagram showing the procedure for pull-down assay. ARPE-19 cells membrane proteins were extracted and mixed with recombinant pentamer protein at 30 μg/ml in citric acid buffer (pH 5.5) and incubated at room temperature for 1 h. Then, the mixtures were adjusted to neutral pH using 1M Tris-HCl buffer. The pentamer in the mixture was pulled down by a pentamer-specific antibody 2–25 along with Protein A/G Magnetic Beads. The pull-down proteins were separated on SDS-PAGE and analyzed by mass spectrometry. (D) 23 membrane proteins were chosen from the list of proteins identified by mass spectrometry assay using membrane protein with extracellular domain as criteria. (TIF) [file ppat.1007914.s001.tif]

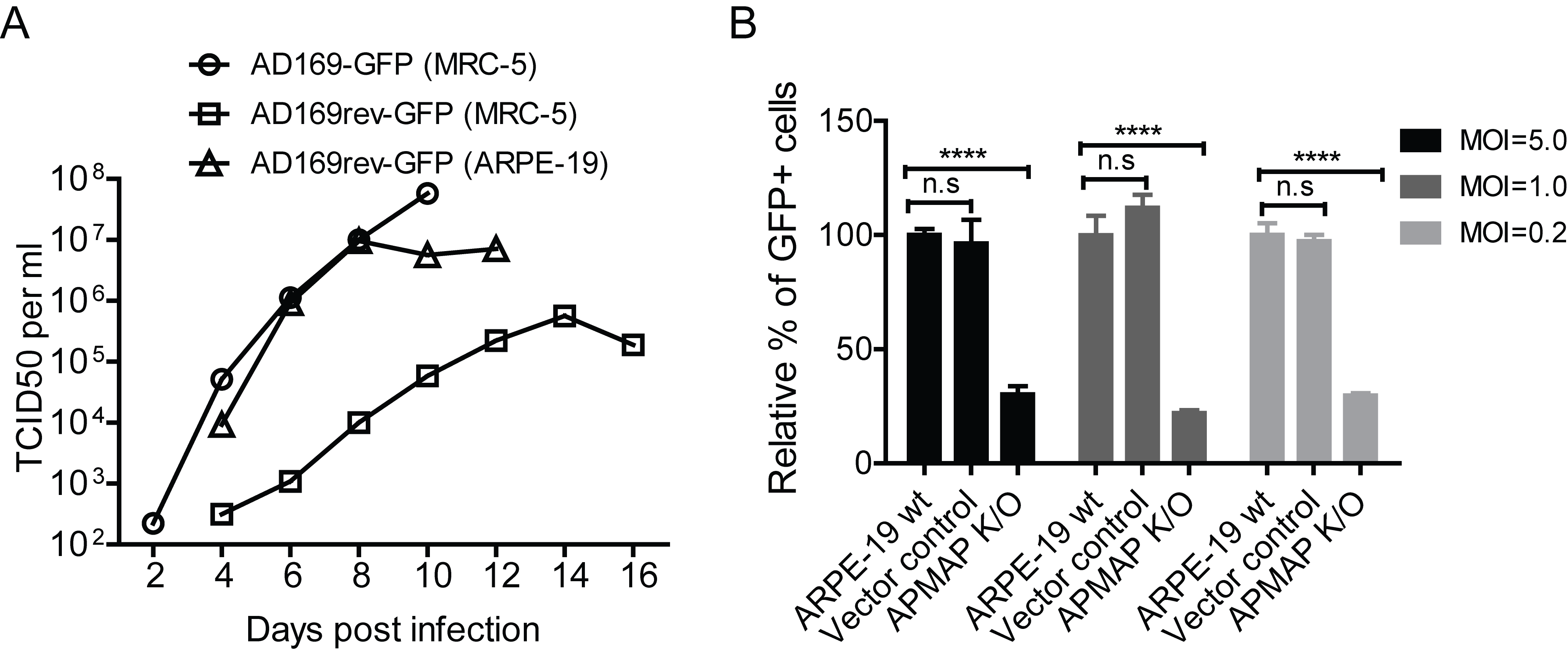

Supplement: S2 Fig — (A) Single step growth curves of AD169-GFP and AD169rev-GFP in MRC-5 or ARPE-19 cells. The infectious viral particles were measured in TCID50 assays. (B) Wildtype ARPE-19, vector control and APMAP K/O cells cultured in 96-well plate were infected with AD169rev-GFP at indicated MOIs. Four replicate wells were infected at each MOI. 72 h later, the plate was read by C.T.L. Immunospot machine to capture images under fluorescence cell mode for GFP. GFP positive cells in each well were counted automatically. The data are shown as relative percentages of the number of GFP positive cells to that of infected wildtype ARPE-19 cells at same MOI. The relative % of GFP+ cells in vector control and APMAP K/O cells were compared individually to that of wildtype ARPE-19 cells at same MOI using unpaired two-tailed student t-test for significance analysis. (TIF) [file ppat.1007914.s002.tif]

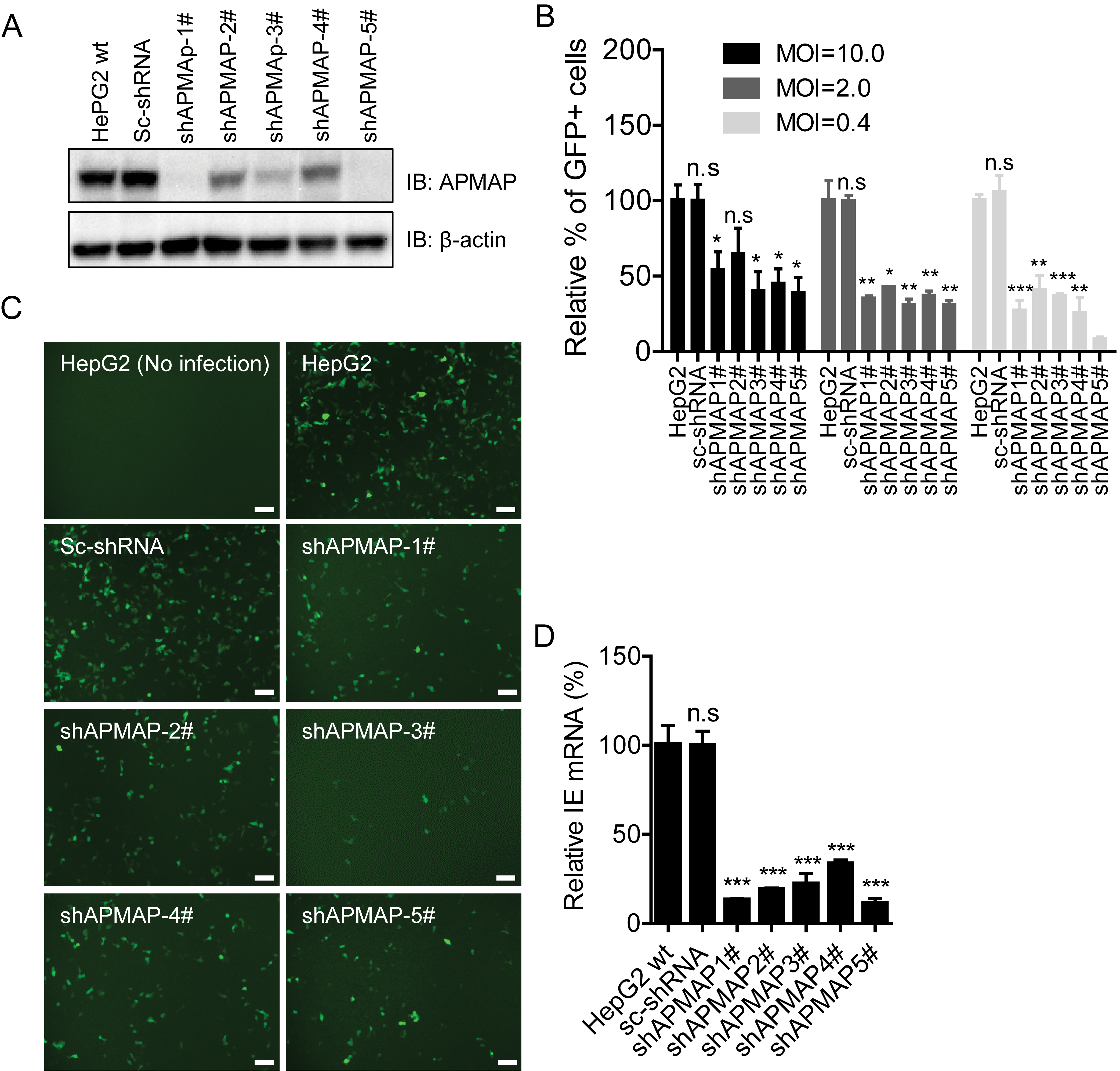

Supplement: S3 Fig — (A) APMAP knockdown in HepG2 cells was achieved by infecting HepG2 cells with lentivirus expressing APMAP-specific shRNA under puromycin selection. APMAP protein expression in the stable knockdown cells were detected by western blot assay using APMAP specific mAb 4F6, β-actin served as loading control. (B-D) Wildtype HepG2 and the APMAP knockdown cells were infected with AD169rev-GFP at indicated MOIs in 96-well plate. (B) The plate was read by C.T.L. Immunospot to capture images under fluorescence cell mode for GFP at 48 h after infection. GFP positive cells in each well were counted automatically using the software. The data are shown as relative percentages of the number of GFP positive cells to that of infected wildtype HepG2 cells. The bars represent means ± SD for four replicate wells. (C) Representative images showing overall GFP positive cells in infected (MOI = 2.0) wildtype and APMAP knockdown HepG2 cells. Images were captured using an Olympus fluorescence microscope. Bar = 100 μm. (D) The cells were collected at 2 days after infection for qRT-PCR detection of viral IE mRNA. GAPDH mRNA served as internal control. Data analysis was performed using the 2-ΔΔCT method. The data are shown as relative percentages of IE mRNA level to that of infected wildtype HepG2 cells. The black bars represent means ± SD for triplicate wells. The relative % of GFP positive cells or relative IE mRNA (%) in sc-shRNA or shAPMAP treated cells were compared individually to that of wildtype HepG2 cells infected at same MOIs using unpaired two-tailed student t-test for significance analysis. (TIF) [file ppat.1007914.s003.tif]

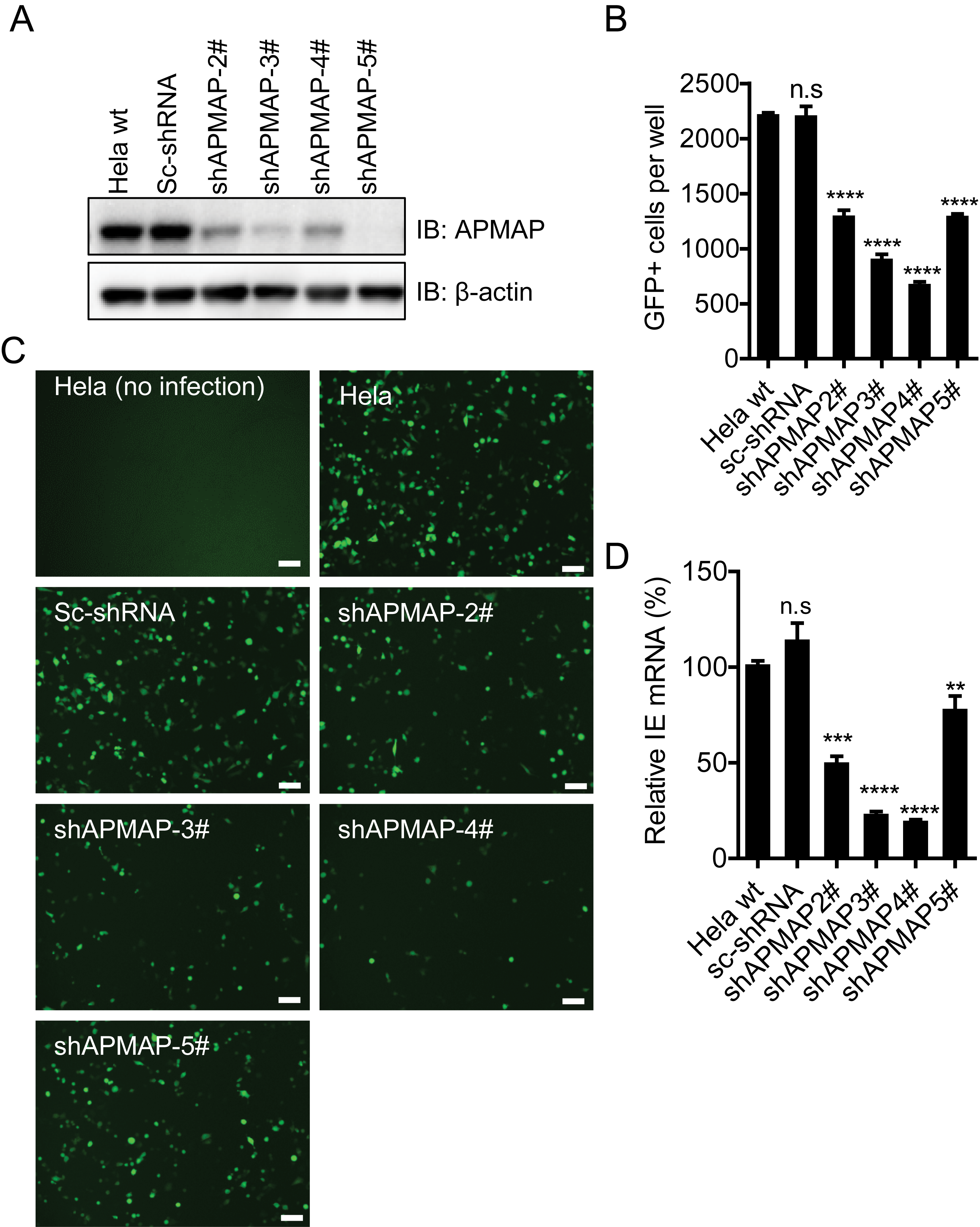

Supplement: S4 Fig — (A) APMAP knockdown in HeLa cells was achieved by infection with lentivirus particles expressing APMAP-specific shRNA under puromycin selection. APMAP protein expression in the stable knockdown cells was detected by western blot assay using APMAP specific mAb 4F6, β-actin served as loading control. (B-D) Wildtype HeLa and the APMAP knockdown cells were infected with AD169rev-GFP (MOI = 1.0) in 96-well plate. (B) The plate was read by C.T.L. Immunospot machine at 48 h after infection and GFP positive cells in each well were counted automatically using the software. The data were shown as the number of GFP positive cells per well. The black bars represent means ± SD for four replicate wells. (C) Representative images showing overall GFP positive cells in wildtype and APMAP knockdown HeLa cells. Images were captured using Olympus fluorescence microscopy. Bar = 100 μm. (D) The cells were collected at 2 days after infection for qRT-PCR detection of viral IE mRNA. GAPDH mRNA served as internal control. Data analysis was performed using the 2-ΔΔCT method. The data are shown as relative percentages of IE mRNA level to that of infected wildtype Hela cells. The black bars represent means ± SD for triplicate wells. The number of GFP positive cells, and relative IE mRNA (%) in sc-shRNA and shAPMAP expressing cells were compared individually to that of wildtype HeLa cells using unpaired two-tailed student t-test for significance analysis. (TIF) [file ppat.1007914.s004.tif]

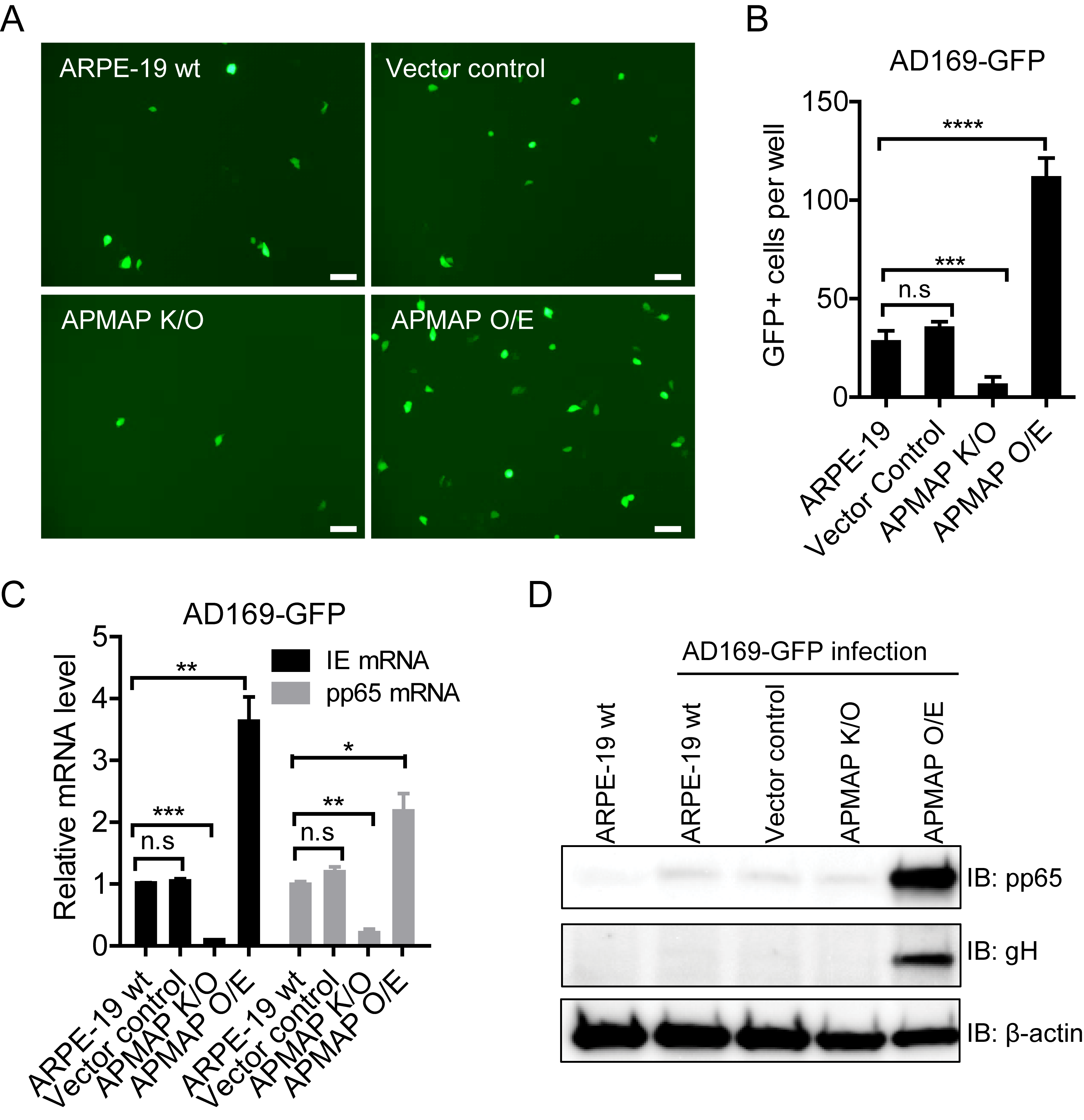

Supplement: S6 Fig — (A) APMAP K/O, O/E and wildtype ARPE-19 cells grow in 96-well plate were infected with AD169-GFP (MOI = 1.0), respectively. The cells were imaged under Fluorescence microscopy to see GFP expression at 2 days post infection using Olympus Fluorescence microscopy. Bar = 100 μm. (B) The plate was read by C.T.L. Immunospot machine at 48 h after infection and GFP positive cells in each well were counted automatically using the software. The data were shown as the number of GFP positive cells per well. The black bars represent means ± SD for four wells. (C) The cells were collected at 2 days after infection for qRT-PCR detection of viral IE and pp65 mRNA. GAPDH mRNA served as internal control. Data analysis was performed using the 2-ΔΔCT method. The data are shown as relative IE or pp65 mRNA level to that of infected wildtype cells. The black bars represent means ± SD for triplicate wells. The number of GFP positive cells, relative IE mRNA and relative pp65 mRNA in vector control, APMAP K/O and APMAP O/E cells were compared individually to that of wildtype ARPE-19 cells using unpaired two-tailed student t-test for significance analysis in S6B and S6C Fig. (D) Determination of HCMV protein expression of AD169-GFP infected cells at 6 days post infection by western blot assay using anti-pp65 and anti-gH antibodies. β-actin served as loading control. (TIF) [file ppat.1007914.s006.tif]

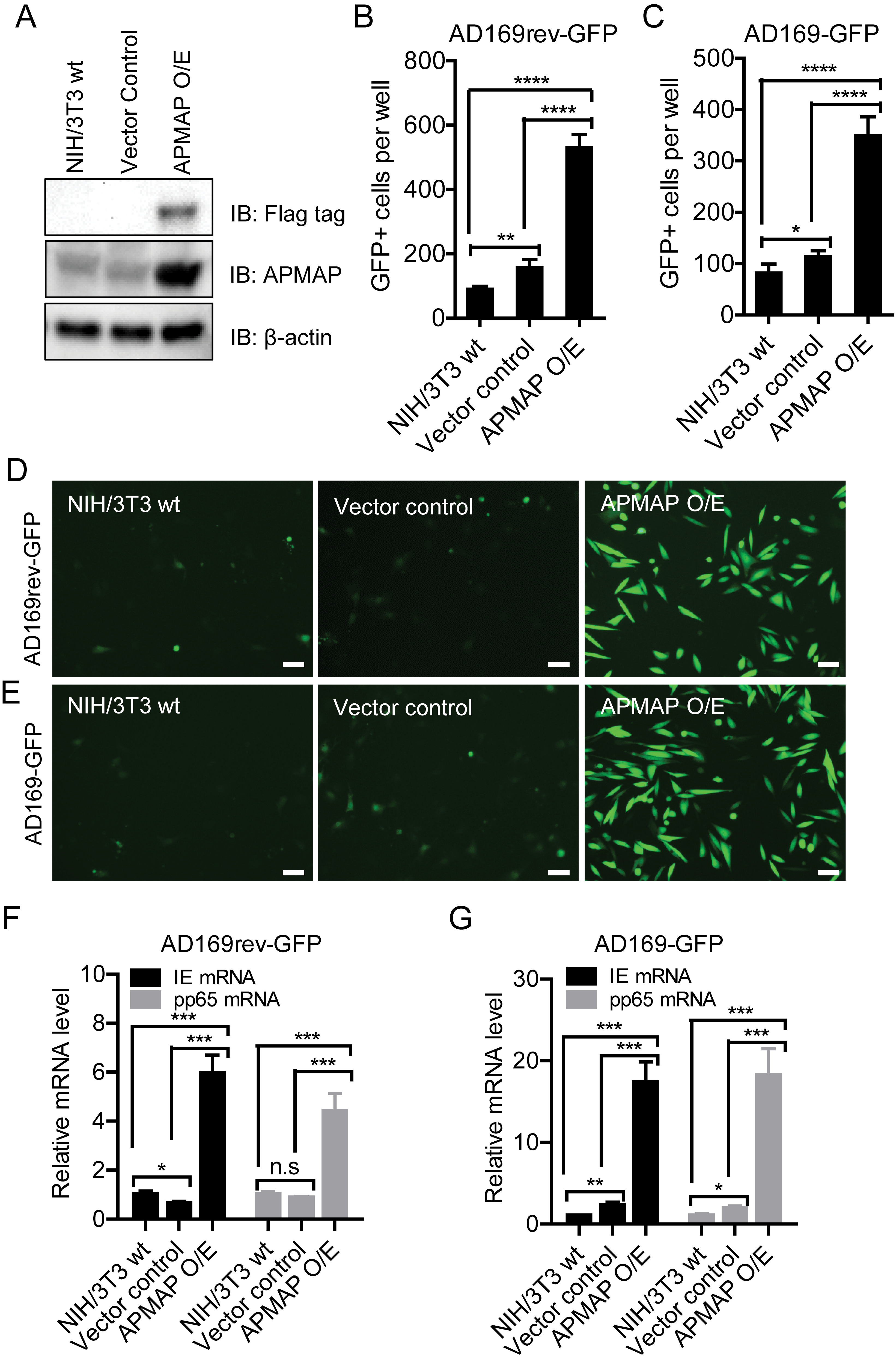

Supplement: S7 Fig — (A) APMAP O/E stable NIH/3T3 cells were established with lentivirus particles carrying APMAP expression cassette with full length sequence with a Myc/Flag tag at its C-terminus. APMAP overexpression in NIH/3T3 cells were confirmed by western blot analysis using mouse anti-Flag tag or anti-APMAP (4F6) antibodies. β-actin served as loading control. (B-F) NIH/3T3 wildtype and APMAP O/E stable cells were infected with AD169rev-GFP and AD169-GFP at MOI = 1.0 in 96-well plate, respectively. (B-C) The plate was read by C.T.L. Immunospot machine to capture images under fluorescence cell mode for GFP at 2 days post infection. GFP positive cells in each well were enumerated automatically using the software. The data were shown as means ± SD of the number of GFP positive cells of four replicate wells. (D-E) Representative images (Bar = 100 μm) showing overall GFP positive cells at day 3 post infection by (D) AD169rev-GFP or (E) AD169-GFP were captured by Olympus fluorescence microscopy. (F-G) The cells were collected at 2 days after infection for qRT-PCR detection of viral IE and pp65 mRNA. GAPDH mRNA served as internal control. Data analysis was performed using the 2-ΔΔCT method. The data are shown as relative IE or pp65 mRNA level to that of infected wildtype cells. The black bars represent means ± SD for triplicate wells. The number of GFP positive cells, relative IE mRNA and relative pp65 mRNA in vector control and APMAP O/E cells were compared individually to that of wildtype NIH/3T3 cells using unpaired two-tailed student t-test for significance analysis in S7B, S7C, S7F and S7G Fig. (TIF) [file ppat.1007914.s007.tif]

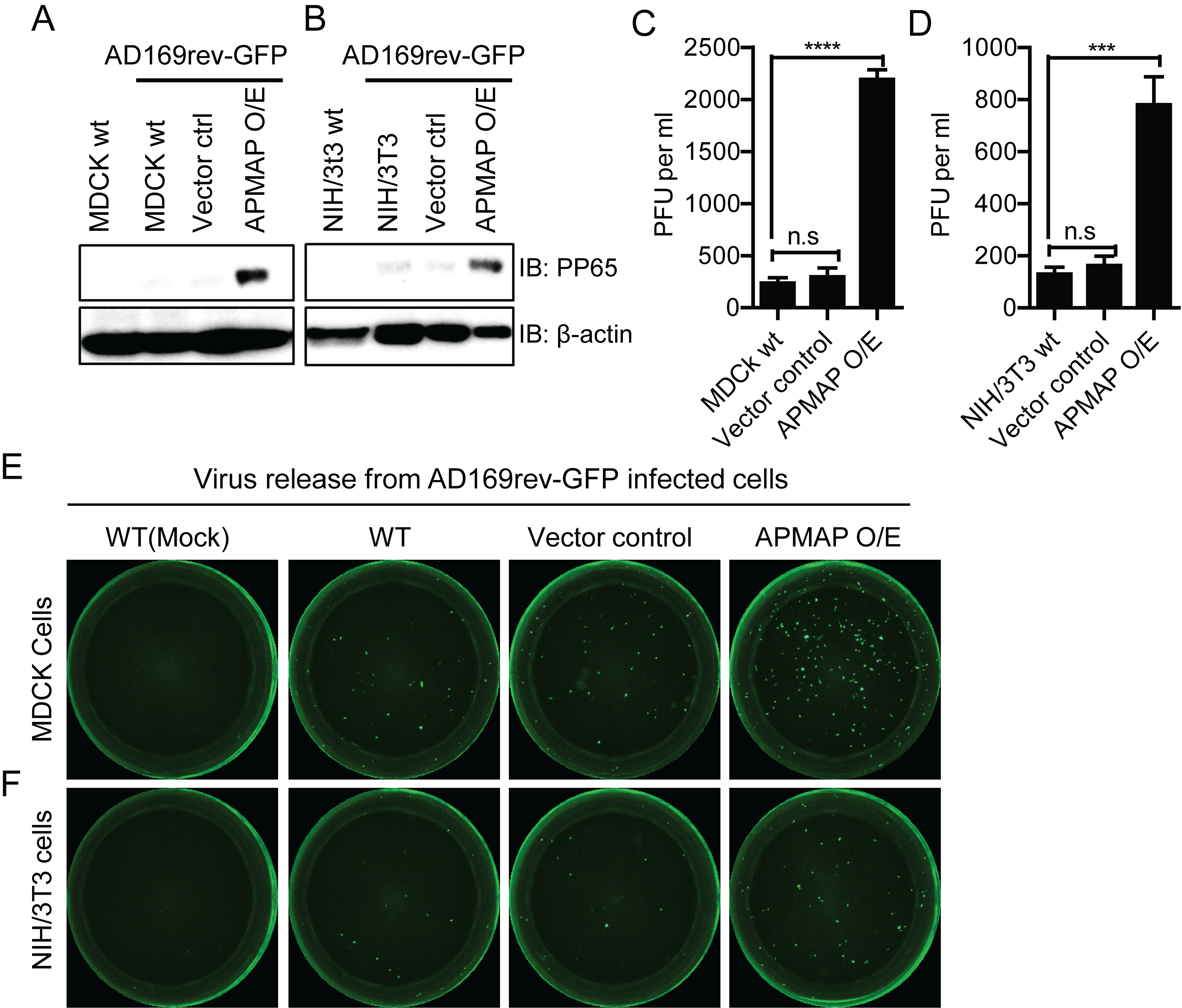

Supplement: S8 Fig — Wildtype, vector control, and APMAP O/E MDCK or NIH/3T3 cells were seeded (1.5×10^5^ cells/well) in a 24-well plate. The next day, the cells were infected with AD169rev-GFP at a MOI = 1.0 for 3 h at 37°C. Virus containing medium was removed and 1 ml of fresh medium was added to each well. After 4 days of culture, the cells were harvested. (A-B) Western blot analysis was performed for detection of viral protein pp65. β-actin served as loading control. The cell culture medium was centrifuged at 10,000×g for 5 mins to remove cell debris. 100 μl supernatant was added to one well of MRC-5 cells grown in a 96-well plate for detection of virus release. 48 h later, images of GFP positive cells among infected MRC-5 cells were captured using a C.T.L. Immunospot machine and counted using the instrument’s software. (C-D) Titer of infectious virion released from infected MDCK and NIH/3T3 cells. One GFP positive cell was counted as one PFU. Black bars represent means ± SD for three replicate wells. The titer of infectious virion produced by vector control and APMAP O/E cells were compared individually to corresponding wiltype cells using unpaired two-tailed student t-test for significance analysis. (E-F) Representative whole-well images of culture medium infected MRC-5 cells. Infection experiments were performed in triplicate wells. Data were representative results of two independent experiments. (TIF) [file ppat.1007914.s008.tif]

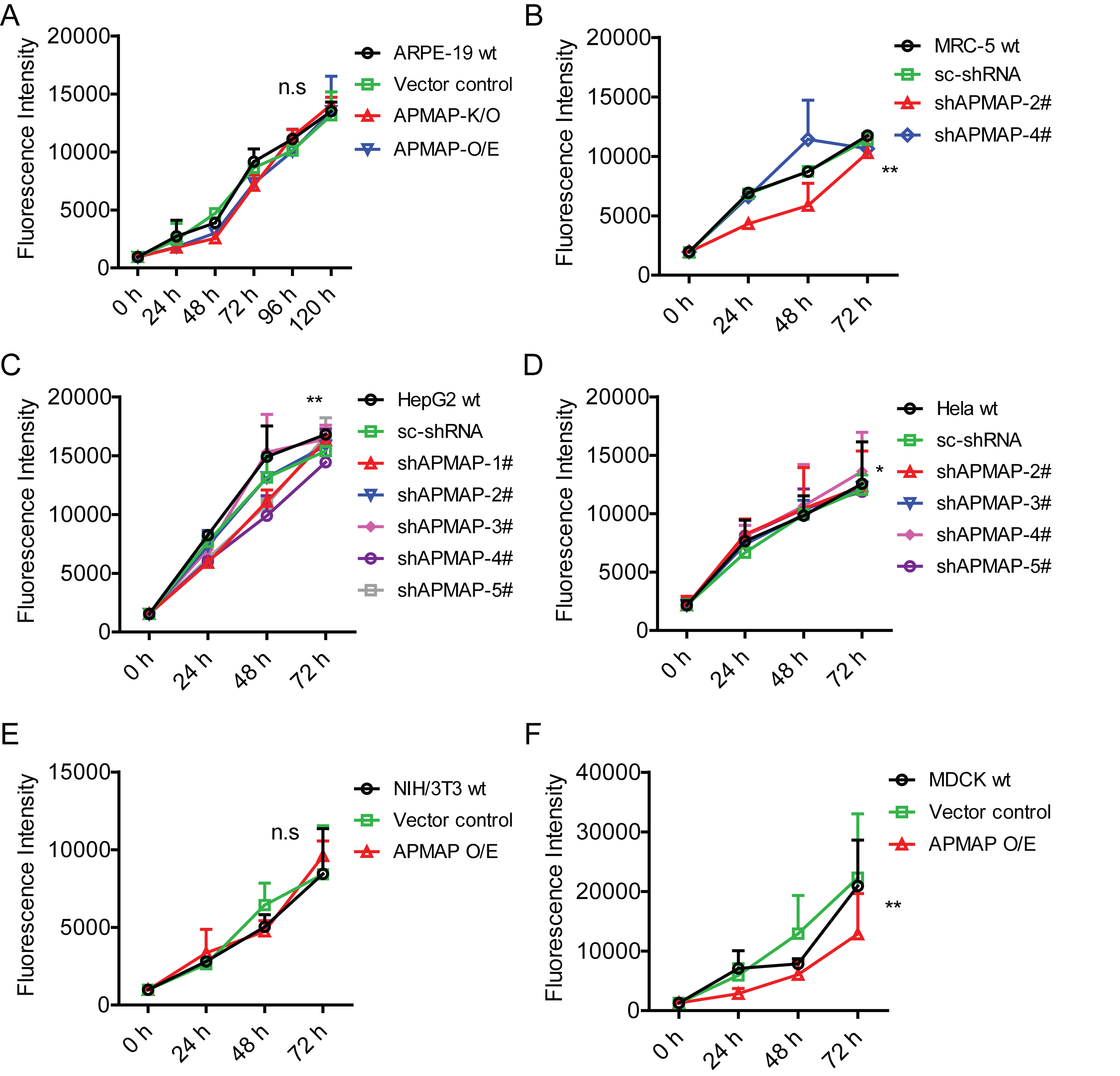

Supplement: S9 Fig — The APMAP K/O, K/D or O/E (A) ARPE-19 cells, (B) MRC-5 cells, (C) HepG2 cells, (D) Hela cells, (E) NIH/3T3 cells and (F) MDCK cells were seeded in 96-well culture plate at 2000 cells per well and cultured for 3–5 days. AlamarBlue solution was added to the cells shortly after seeding and then every 24 h. Fluorescence intensity was measured at 4 h post addition of alamarBlue as instructed by the manufacture. At each time point, 6 replicate wells were detected for each cell line. Data were shown as mean ± SD of fluorescence intensity. Two-way ANOVA analyses was used to compare the growth rates of vector control, APMAP K/O (K/D), or APMAP O/E cells with the corresponding wildtype cells. (TIF) [file ppat.1007914.s009.tif]

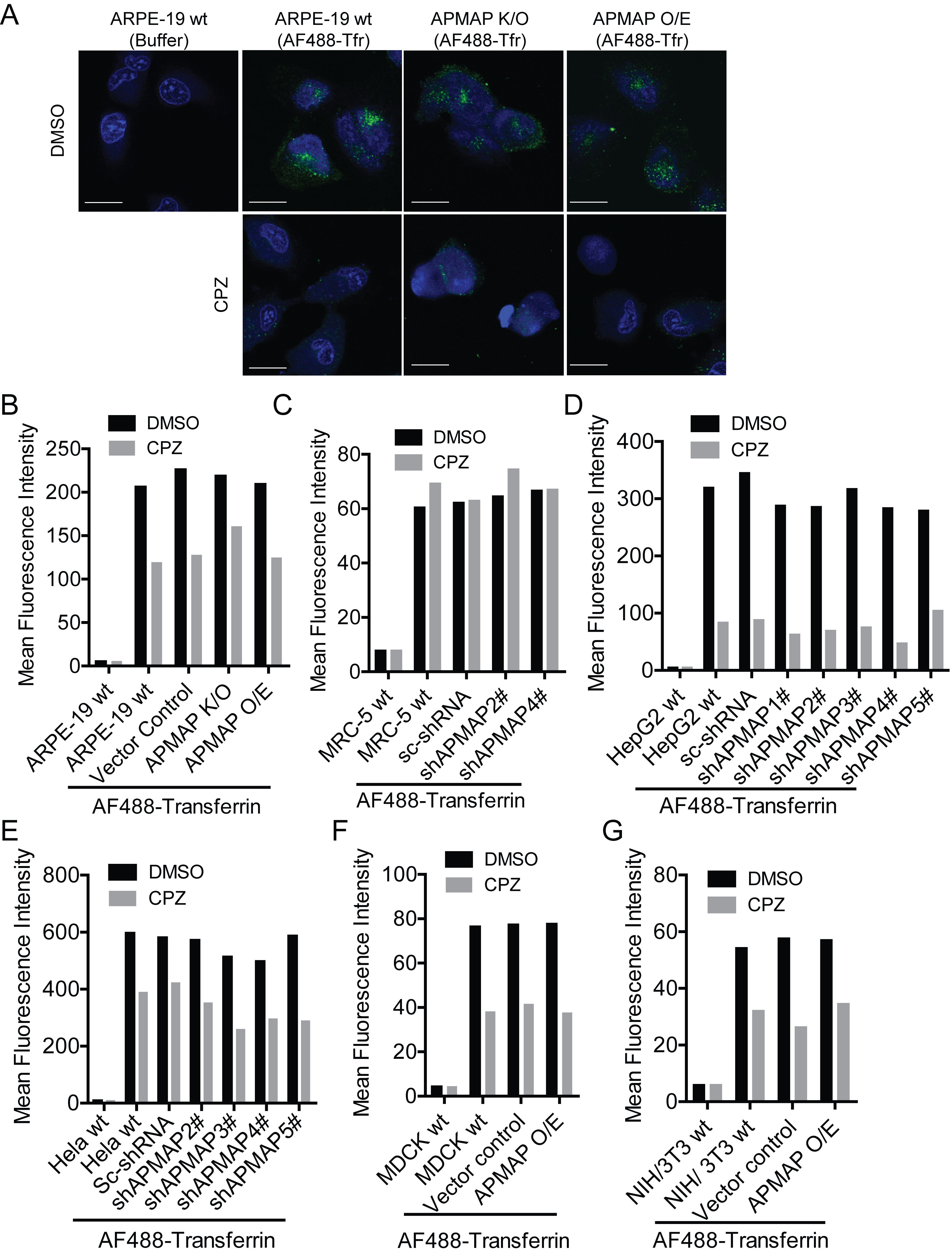

Supplement: S10 Fig — (A) ARPE-19 wt, APMAP K/O and APMAP O/E cells grown in chamber slides were pre-treated with 30 μm chlorpromazine (CPZ) or equal volume of solvent (DMSO) in serum free medium for 1h. Old medium was removed. 100 μl/well of AF488-Tfr (0.1 mg/ml) in fresh medium was incubated with the cells at 37°C for 30 min. The cells were washed once with citrate buffer (40 mM citric acid, 10 mM KCl, 135 mM NaCl, pH 3.0) and 3 times with PBS to remove cell surface AF488-Tfr before fixation with 4% paraformaldehyde and nucleus staining with DraQ5. Pictures were taken using a LEIKA confocal microscopy. Bar = 20 μm. (B-G) Quantification of AF488-Tfr uptake in APMAP K/O K/D or O/E (B) ARPE-19 cells, (C) MRC-5 cells, (D) HepG2 cells, (E) Hela cells, (F) MDCK cells and (G) NIH/3T3 cells by flow cytometry assay. Cells grown in 12-well plate were pre-treated with CPZ and incubated with AF488-Tfr as described in (A). After washing with citrate buffer to remove cell surface AF488-Tfr, the cells were suspended by treatment with trypsin and detected using on a Guava easycyte HT machine. Data were shown as mean fluorescent intensities of green signal for each samples. (TIF) [file ppat.1007914.s010.tif]

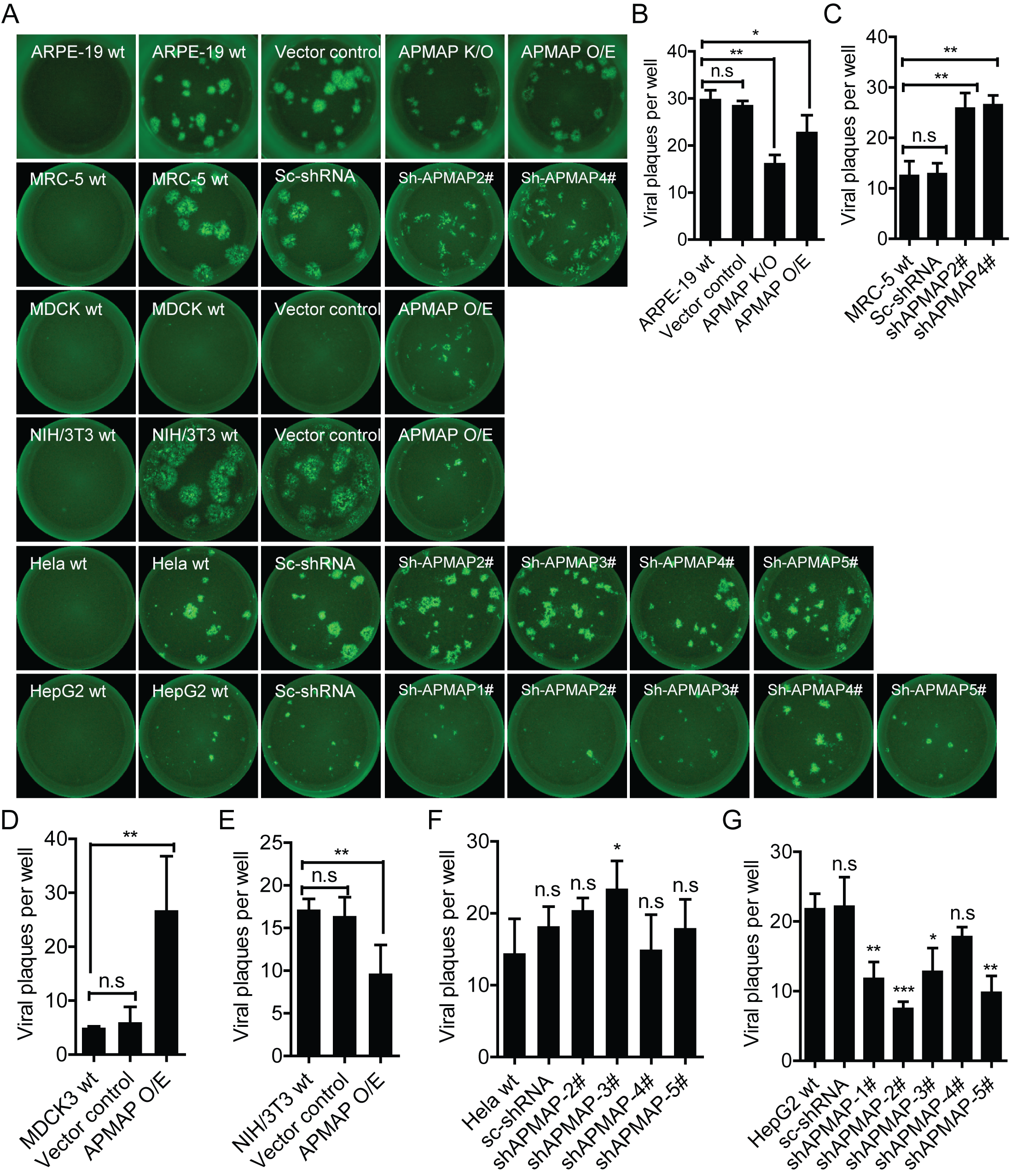

Supplement: S11 Fig — The cells (1.4×10^4^ cells per well) were seeded in 96-well plate one day ahead infection. Cells with about 95% confluency were infected with HSV-2 GFP reporter virus at about 60 PFU/well for ARPE-19 cells, 300 PFU/well for MRC-5, HepG2 and Hela cells, 1500 PFU/well for NIH/3T3 and MDCK cells with at least three replicate wells. Medium was removed at 48 h post infection. The plate were imaged at same exposure time and gain using Immunospot 7.0 Pro FluoRo-X suite for GFP detection on a C.T.L. Immunospot machine. (A) Representative single-well images of infected cells. (B-G) GFP positive viral plaques in the wells were counted manually. Data were shown as mean ± SD of the number of GFP positive viral plaques. The number of GFP positive cells in vector control, APMAP K/O, K/D or O/E cells were all compared individually to that of corresponding wildtype cells using unpaired two-tailed student t-test for significance analysis. (TIF) [file ppat.1007914.s011.tif]

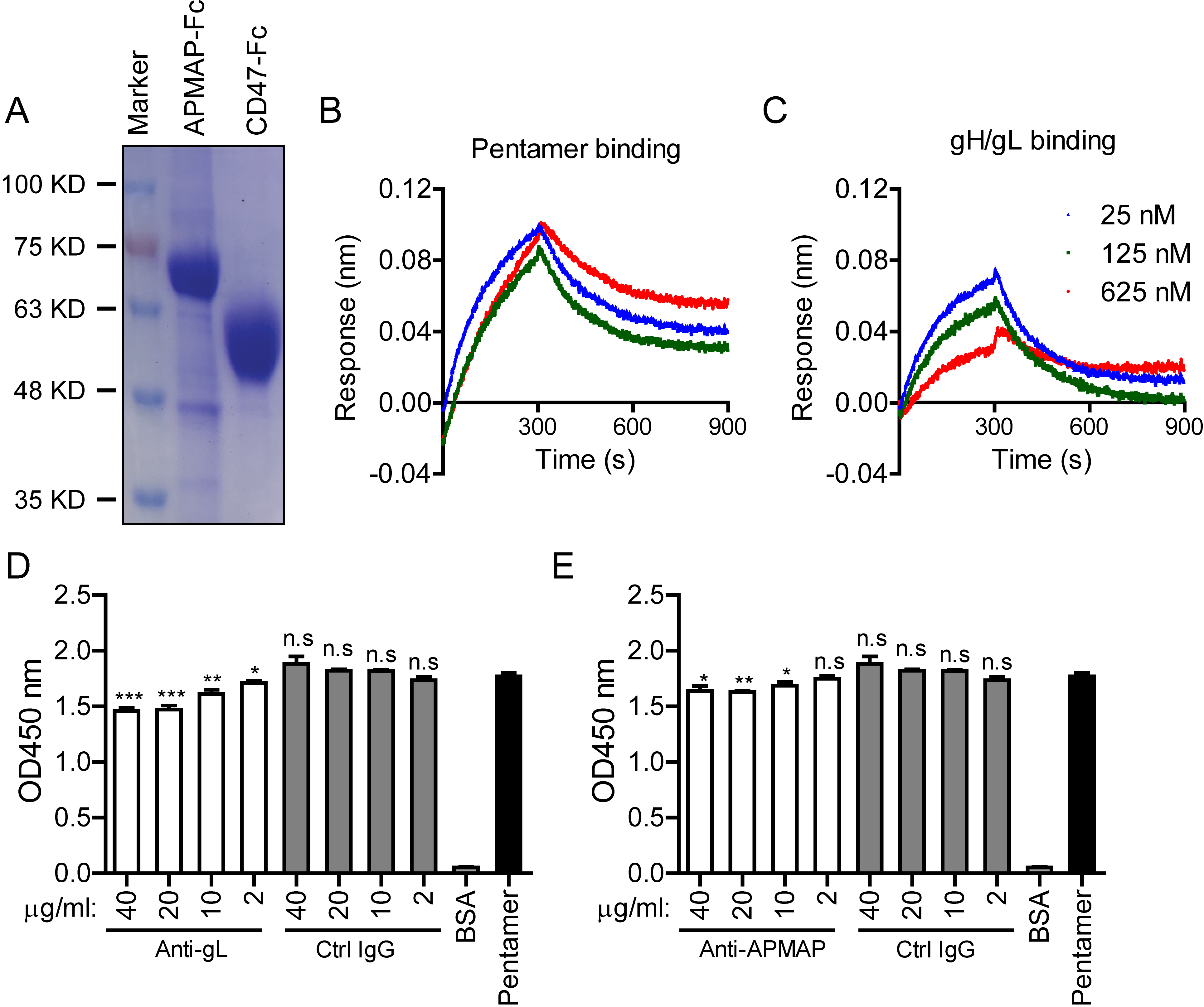

Supplement: S12 Fig — (A) 10 μg of purified APMAP-Fc and a control CD47-Fc protein analyzed by SDS-PAGE and coomassie blue staining assay. (B-C) The binding of (B) pentamer and (C) gH/gL dimer to APMAP-Fc at indicated concentrations were detected by biolayer interferometry (BLI) assay using protein A sensors. The binding of kinetic buffer to APMAP-Fc loaded sensor were used as reference and subtracted before data analysis. (D-E) Inhibition of soluble pentamer binding to APMAP by gL or APMAP specific rabbit polyclonal antibodies. Costar 96-well high binding plates were incubated with APMAP-Fc (4 μg/ml in PBS, 50 μl/well) overnight at 4°C. Unbound APMAP-Fc was removed. The plate was blocked with 5% non-fat milk for 1 h. Soluble pentamer (final concentration 5 μg/ml) was incubated with purified rabbit anti-gL polyclonal IgGs, rabbit anti-APMAP polyclonal IgGs or rabbit anti-HSV polyclonal IgGs (as control IgG) at indicated concentrations in low pH buffer (150 mM Citric acid, 50 mM NaCl, pH 5.5) at room temperature for 30 mins. Pentamer incubated without antibody served as positive control. 50 μl/well of the mixture was added to APMAP coated plate and incubated at 37°C for 2 h. BSA diluted in low pH buffer served as negative control. Wells were tested in triplicate for each concentration. The plate was washed with PBST five times. HRP conjugated anti-His tag antibody (1:2000 dilution) that recognizes the gH subunit were added to plate for detection of soluble pentamer. The plate was washed with PBST five times then developed using TMB substrate mixture. Absorbance at 450nm was recorded on a Molecular Devices Spectra Max M4. Data are presented as mean values ± standard deviation (SD). The OD450nm values of all samples were compared individually to that of pentamer-only control using the unpaired two-tailed student t-test for significance analysis. (TIF) [file ppat.1007914.s012.tif]

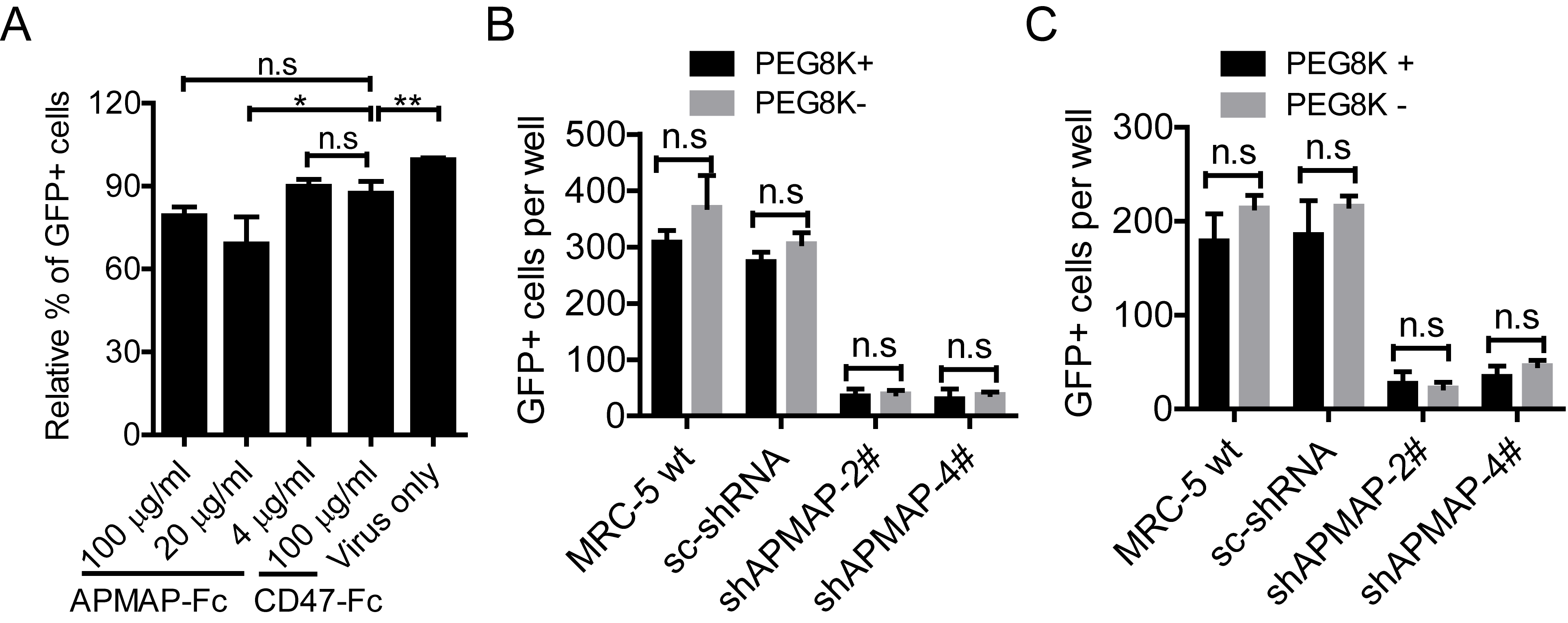

Supplement: S13 Fig — (A) 50 μl APMAP-Fc or CD47-Fc control were mixed with equal volume of AD169rev-GFP (about 100 PFU/well) at indicated concentrations and incubated at 37°C for 30 min before adding to pre-seeded ARPE-19 cells in 96-well plate. 2 hours later the virus mixtures were replaced with fresh medium. The cells were continue cultured for 3 days before quantitation of GFP positive cells in each well. Pairs of samples were compared inividually using the unpaired two-tailed student t-test for significance analysis. (B-C) Wildtype, sc-shRNA control and the APMAP K/D MRC-5 cells were seeded in 96-well plate 1 day before. (B) AD169rev-GFP and (C) AD169-GFP were added to the cells at a MOI = 1.0 and cultured at 37°C for 1 h. The uninfected virus was removed by washing with warm PBS for 2 times. Then, the cells were treated with150 μl/well of pre-warmed 44% (W/V) PEG-8K for 1 min and followed by washing with 200 μl/well of warm PBS for 4 times to remove PEG-8K. After that, fresh medium was added to cells and the plate was continue cultured for 48 h before read by C.T.L. Immunospot machine to capture images under fluorescence cell mode for GFP. The number of GFP positive cells in each well was counted using the software. The data are shown as means ± SD of the number of GFP positive cells in four replicate wells. The number of GFP positive cells in PEG8K treated cell lines were compared to corresponding non-treated cell lines using the unpaired two-tailed student t-test for significance analysis. (TIF) [file ppat.1007914.s013.tif]
