# Supplementary material for: Identification of adipocyte plasma membrane-associated protein as a novel modulator of human cytomegalovirus infection
Source: PLoS Pathog. 2019 Jul 29;15(7):e1007914. doi: 10.1371/journal.ppat.1007914 (PMC6687193; doi:10.1371/journal.ppat.1007914)
Supplement: S1 Table — (DOCX) [file ppat.1007914.s014.docx]

**S1 Table.** Sequences of sgRNA and shRNA.

| Name | Sequence (5’-3’) |
| --- | --- |
| Sc-sgRNA | CACCGGCACTCACATCGCTACATCA |
| COMT-sgRNA-1 | CACCGCACCAGCCCTCCGTGCTGCGT |
| COMT-sgRNA-1 | CACCGTTGCTTTTAACTTTGACAGGT |
| VAMP5-sgRNA-1 | CACCGTTCTGTGTAGTCTTGTTGAGT |
| VAMP5-sgRNA-2 | CACCGAACAAGACTACACAGAACCGT |
| SCARB2-sgRNA-1 | CACCGGTGTTAAGGAATGGTACTGGT |
| SCARB2-sgRNA-2 | CACCGTTCCTCAGGGAGATCATCGGT |
| VAMP2-sgRNA-1 | CACCGGACATCATGAGGGTGAACGGT |
| VAMP2-sgRNA-2 | CACCGATGAGGGTGAACGTGGACAGT |
| CLPTM1-sgRNA-1 | CACCGAAGTAAACGTGGATGTAGAGT |
| CLPTM1-sgRNA-2 | CACCGCTTCTGCCGGGGGTCTGGGGT |
| TMEM33-sgRNA-1 | CACCGTGCTCTTACCAGTGCTCTGGT |
| TMEM33-sgRNA-2 | CACCGCAAAGAGAATAACAAGACTGT |
| FAM210B-sgRNA-1 | CACCGGCCAGGCCACGGGGACAACGT |
| FAM210B-sgRNA-2 | CACCGAAAAGATTTTTCAAGAGTAGT |
| TMEM109-sgRNA-1 | CACCGGAGATGGCCCACAACACTTGT |
| TMEM109-sgRNA-2 | CACCGTGGCCTTCTTTGCTCTGTCGT |
| TMTC3-sgRNA-1 | CACCGTAAAAATAATGCCAAACTTGT |
| TMTC3-sgRNA-2 | CACCGAAGTTTTTAAAGGTGTAGAGT |
| TMEM43-sgRNA-1 | CACCGGGGATGTTTGTGGGGCTCAGT |
| TMEM43-sgRNA-2 | CACCGGGTTGCCGTCTTCAATGCGGT |
| SCAMP3-sgRNA-1 | CACCGGAGGCAATGGGGCAGGGGCGT |
| SCAMP3-sgRNA-2 | CACCGGCTGTGGCTGCTGCAGCTGGT |
| TMEM2-sgRNA-1 | CACCGCGTTTCTCTTTCAGGATTCGT |
| TMEM2-sgRNA-2 | CACCGAAACCTGGAGACCAGATTGGT |
| TM9SF2-sgRNA-1 | CACCGGCTTTCTACCTGCCCGGCCGT |
| TM9SF2-sgRNA-2 | CACCGTTCACAAATAGTTCTATTTGT |
| PGRMC1-sgRNA-1 | CACCGGGCCGTATGGGGTCTTTGCGT |
| PGRMC1-sgRNA-2 | CACCGCAAAATGTGGCAAGGCCCCGT |
| SCAMP4-sgRNA-1 | CACCGATGAGGTTGACGCCGAGGGGT |
| SCAMP4-sgRNA-2 | CACCGCATTGCCTGCCTGGCCTGGGT |
| APMAP-sgRNA-1 | CACCGCAGCATCAAGAAGGTCACTGT |
| APMAP-sgRNA-2 | CACCGAAGAACCCCCGCTCTTGCTGT |
| TM9SF3-sgRNA-1 | CACCGTAAAGAGGAAGTTGTCTTAGT |
| TM9SF3-sgRNA-2 | CACCGCAATTTCACAGTAAGTGGCGT |
| VAMP7-sgRNA-1 | CACCGTCATTACATCTGCCAAGACGT |
| VAMP7-sgRNA-2 | CACCGTTCAGAAAATTAAAGGCTCGT |
| VMP1-sgRNA-1 | CACCGCCAAATCCTTTATTTGATCGT |
| VMP1-sgRNA-2 | CACCGACACTTTCTGGTACCTTTTGT |
| TMEM41B-sgRNA-1 | CACCGTACTGAGAAATATAGAGCCGT |
| TMEM41B-sgRNA-2 | CACCGATTTCTCAGTATACTCTCAGT |
| LMAN2-sgRNA-1 | CACCGCCCAGTCTTTGAGGAAGCAGT |
| LMAN2-sgRNA-2 | CACCGACTTCAAAGTCCACGGCACGT |
| SCAMP1-sgRNA-1 | CACCGGTTGTGTATTGGGTACATTGT |
| SCAMP1-sgRNA-2 | CACCGCGCTTAAGAAGTTCAGCTTGT |
| VAPA-sgRNA-1 | CACCGGAAGACTACAGCACCTCGCGT |
| VAPA-sgRNA-2 | CACCGCCACCAAACACTTCAGATAGT |
| shAPMAP-1 | ACCGGAGTCCATAGCACATAT |
| shAPMAP-2 | TATCTGCCATGAGGAACATAA |
| shAPMAP-3 | TACTGTGACCAGGGAAGTAAA |
| shAPMAP-4 | ATATATAACGTGCGGTCATAC |
| shAPMAP-5 | CTTGGTGTTCTGCATCCAAAT |
| Sc-shRNA | CCTAAGGTTAAGTCGCCCTCG |
